# Dihydroceramide accumulation mediates cytotoxic autophagy of cancer cells via autolysosome destabilization

**DOI:** 10.1080/15548627.2016.1213927

**Published:** 2016-09-16

**Authors:** Sonia Hernández-Tiedra, Gemma Fabriàs, David Dávila, Íñigo J. Salanueva, Josefina Casas, L. Ruth Montes, Zuriñe Antón, Elena García-Taboada, María Salazar-Roa, Mar Lorente, Jesper Nylandsted, Jane Armstrong, Israel López-Valero, Christopher S. McKee, Ana Serrano-Puebla, Roberto García-López, José González-Martínez, José L. Abad, Kentaro Hanada, Patricia Boya, Félix Goñi, Manuel Guzmán, Penny Lovat, Marja Jäättelä, Alicia Alonso, Guillermo Velasco

**Affiliations:** a Department of Biochemistry and Molecular Biology I, School of Biology, Complutense University, Madrid, Spain; b Instituto de Investigaciones Sanitarias San Carlos (IdISSC), Madrid, Spain; c Research Unit on BioActive Molecules (RUBAM), Departments of Biomedicinal Chemistry, Institute for Advanced Chemistry of Catalonia (IQAC-CSIC), Barcelona, Spain; d Biofisika Institute (UPV/EHU, CSIC), and Departamento de Bioquímica, Universidad del País Vasco, Barrio Sarriena s/n, Leioa, Spain; e Unit of Cell Death and Metabolism, Center for Autophagy, Recycling and Disease, Danish Cancer Society Research Center (DCRC), Copenhagen, Denmark; f Dermatological Sciences, Institute of Cellular Medicine, Newcastle University, Newcastle-upon-Tyne, UK; g Faculty of Applied Sciences, University of Sunderland, Sunderland, UK; h Departament of Cellular and Molecular Biology, Centro de Investigaciones Biológicas, CSIC, Madrid, Spain; i Department of Biochemistry and Cell Biology, National Institute of Infectious Diseases, Shinjuku-ku, Tokyo, Japan; j Centro de Investigación Biomédica en Red Sobre Enfermedades Neurodegenerativas, Instituto Ramón y Cajal de Investigación Sanitaria, Madrid, Spain, Instituto Universitario de Investigación Neuroquímica, Complutense University, Madrid, Spain

**Keywords:** autophagy, cancer, cannabinoids, cell death, sphingolipids

## Abstract

Autophagy is considered primarily a cell survival process, although it can also lead to cell death. However, the factors that dictate the shift between these 2 opposite outcomes remain largely unknown. In this work, we used Δ^9^-tetrahydrocannabinol (THC, the main active component of marijuana, a compound that triggers autophagy-mediated cancer cell death) and nutrient deprivation (an autophagic stimulus that triggers cytoprotective autophagy) to investigate the precise molecular mechanisms responsible for the activation of cytotoxic autophagy in cancer cells. By using a wide array of experimental approaches we show that THC (but not nutrient deprivation) increases the dihydroceramide:ceramide ratio in the endoplasmic reticulum of glioma cells, and this alteration is directed to autophagosomes and autolysosomes to promote lysosomal membrane permeabilization, cathepsin release and the subsequent activation of apoptotic cell death. These findings pave the way to clarify the regulatory mechanisms that determine the selective activation of autophagy-mediated cancer cell death.

## Introduction

Macroautophagy, hereafter named autophagy, is a highly conserved cellular process in which cytoplasmic materials, including organelles, are sequestered into double-membrane compartments, phagophores, that mature into autophagosomes; the cargo is subsequently delivered to lysosomes for degradation and recycling.^1-3^ In many cellular settings, triggering of autophagy relies on the inhibition of MTORC1 (mechanistic target of rapamycin [serine/threonine kinase] complex 1), an event that promotes the activation (de-inhibition) of several ATG (autophagy-related) proteins involved in the initial phase of phagophore formation.^1-3^ The membrane source from which autophagosomes are derived is still debatable, as it has been proposed that it could be derived either from *de novo* synthesized lipids or generated by vesicle budding from the endoplasmic reticulum (ER), Golgi apparatus or endosomes,^4,5^ or the plasma membrane.^6^ In particular, an ER-derived structure termed the omegasome has been proposed as an origin of the phagophore membrane.^5,7^ Enlargement of this compartment to form the autophagosome requires the participation of 2 ubiquitin-like conjugation systems, one involving the conjugation of ATG12 (autophagy-related 12) to ATG5 (autophagy-related 5), and the other of phosphatidylethanolamine to MAP1LC3/LC3 (microtubule-associated protein 1 light chain 3).^2^ The final outcome of the activation of the autophagy program is highly dependent on the cellular context and the strength and duration of the stress-inducing signals. Thus, autophagy plays an important role in cellular homeostasis and is considered primarily a cell-survival mechanism, for example in situations of nutrient deprivation.^8-11^ However, stimulation of autophagy can also have a cytotoxic effect. For example, several anticancer agents activate autophagy-associated cell death.^8-10,12^ However, the molecular mechanisms that determine the outcome of autophagy activation for the survival or death of cancer cells remain to be clarified.

Δ^9^-Tetrahydrocannabinol (THC), the main active component of *Cannabis sativa*,^13,14^ exerts a wide variety of biological effects by mimicking endogenous substances—the endocannabinoids anandamide^15^ and 2-arachidonoylglycerol (2-AG)^16,17^ that engage specific cell-surface G protein-coupled cannabinoid receptors.^14^ So far, 2 major cannabinoid-specific receptors, CNR1/CB_1_ (cannabinoid receptor 1 [brain]) and CNR2/CB_2_ (cannabinoid receptor 2 [macrophage]), have been cloned and characterized from mammalian tissues.^18,19^ Cannabinoid administration curbs the growth of several genetic and xenograft models of cancer in rats and mice, and therefore these compounds are considered a novel family of potential anticancer agents.^20^ The mechanism of cannabinoid anticancer action relies, at least largely, on the ability of these agents to stimulate autophagy-mediated cancer cell death.^20^ Thus, THC binds cannabinoid receptors, which leads to the stimulation of *de novo* sphingolipid synthesis and the subsequent activation of an endoplasmic reticulum (ER) stress-related signaling route that involves the upregulation of the transcriptional co-activator NUPR1/p8 (nuclear protein 1, transcriptional regulator) and its effector TRIB3 (tribbles pseudokinase 3).^20-23^ The stimulation of this pathway promotes in turn autophagy via TRIB3-mediated inhibition of the AKT (thymoma viral proto-oncogene)-MTORC1 axis, which is indispensable for the pro-apoptotic and antitumoral action of cannabinoids.^24,25^

In this study, we have investigated the molecular mechanism underlying the activation of autophagy-mediated cancer cell death by comparing the effects of THC treatment and nutrient deprivation, 2 autophagic stimuli that produce opposite effects on the regulation of cancer cell survival/death. Using this experimental model, we found that treatment with THC—but not exposure to nutrient deprivation—leads to an alteration of the balance between different molecular species of ceramides and dihydroceramides in the microsomal (endoplasmic reticulum-enriched) fraction of cancer cells. Moreover, our findings support the hypothesis that such modification can be transmitted to autophagosomes and autolysosomes, where it can promote the permeabilization of the organellar membrane, the release of cathepsins to the cytoplasm and the subsequent activation of apoptotic cell death.

## Results

### THC-induced, but not nutrient deprivation-induced, autophagy relies on the stimulation of sphingolipid biosynthesis

**Figure 1.**
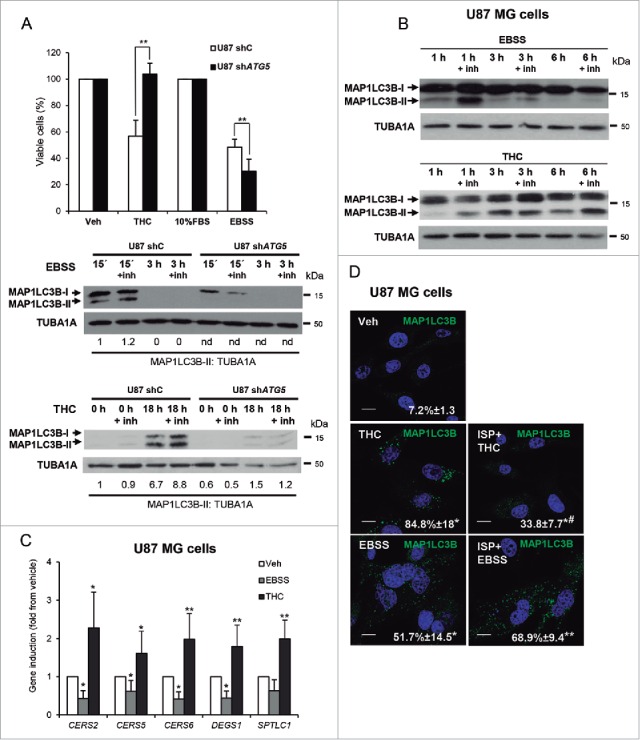
THC, but not nutrient deprivation, -induced autophagy relies on the stimulation of sphingolipid biosynthesis. (A) Upper panel: Effect of THC (4 µM, 18 h) and incubation with EBSS (18 h) on the number of U87MG cells stably transfected with control (shC) or *ATG5*-selective (sh*ATG5*) shRNAs as estimated by the MTT test (n = 4; mean ± s.d; **, *P* < 0.01 from THC-treated or EBSS-incubated U87 shC cells). Lower panel: Effect of THC (4 µM) and incubation with EBSS on the induction of autophagy (as determined by MAP1LC3B-II lipidation in the presence of E64d, 10 μM; and pepstatin A, 10 μg/ml [+inh]) of U87 cells stably transfected with control (U87 shC) or *ATG5*-selective (sh*ATG5*) shRNAs (n = 3, a representative experiment is shown). *ATG5* mRNA levels (as determined by real-time quantitative PCR) were reduced by 85 ± 3% on U87sh*ATG5* cells when compared with U87shC cells; (n = 4). Values in the bottom of the western blots correspond to the fold change in the MAP1LC3B-II to TUBA1A ratio relative to shC U87MG cells at the initial time point of the treatments. Nd, nondetectable. (B) Effect of THC (4 µM, 1 h, 3 h and 6 h) and incubation with EBSS (i.e., nutrient deprivation, 1, 3 and 6 h) on the induction of autophagy (as determined by MAP1LC3B-II lipidation in the presence of E64d, 10 μM; and pepstatin A, 10 μg/ml [+inh]) of U87MG cells (n = 3, a representative experiment is shown). (C) Effect of THC (4 µM; 3 h) on the mRNA levels (as determined by quantitative real-time PCR) of different enzymes involved in sphingolipid biosynthesis (*CERS2*; *CERS5*; *CERS6* (ceramide synthase 2, 5 and 6), *DEGS1/*dihydroceramide desaturase (delta[4]-desaturase, sphingolipid 1) and *SPTLC1* (serine palmitoyltransferase long chain base subunit 1) of U87MG cells (n = 5; *, *P* < 0.05; **, *P* < 0.01 from Veh-treated cells). (D) Effect of THC (4 µM), ISP-1 (5 µM) and incubation with EBSS on autophagy (18 h) (as determined by MAP1LC3B immunostaining). Note that incubation with ISP-1 prevents THC but not starvation-induced autophagy of U87MG cells. Values correspond to the percentage of cells with MAP1LC3B dots relative to the total cell number of cells ± s.d; n = 3. *, *P* < 0.05; **, *P* < 0.01 from Veh-treated cells and ^#^, *P* < 0.05 from THC- and EBSS-treated cells. Bar: 20 μm.

As a first approach to investigate the molecular mechanisms responsible for the activation of autophagy-mediated cancer cell death we analyzed the effect of 2 different stimuli, namely nutrient deprivation and THC treatment, that trigger cytoprotective and cytotoxic autophagy, respectively. We found that genetic inhibition of the autophagy essential gene *ATG5* in both U87MG cells and oncogene-transformed mouse embryonic fibroblasts (MEFs) prevented THC-induced cell death while it further diminished the nutrient deprivation-induced decrease in cell viability (Fig. 1A and Fig. S1A), thus supporting the notion that stimulation of autophagy may play a dual role in the regulation of cancer cell survival.

After confirming that incubation with EBSS and treatment with THC led to an increase in the accumulation of MAP1LC3B-positive dots in U87MG cells (Fig. S1B) we analyzed the ability of these 2 stimuli to enhance the autophagic flux in U87MG cells. To this aim, we performed the treatments in the presence or the absence of the lysosomal proteases inhibitors E64d and pepstatin A (+ inh); upon stimulation of dynamic autophagy and in the presence of these inhibitors there is a blockade of the autophagic flux and therefore an enhanced accumulation of proteins present in the autophagosomes, and specifically of the lipidated and autophagosome-associated form of MAP1LC3, MAP1LC3-II. Of note, incubation with EBSS induced only an early and transient increase in the autophagic flux (EBSS led to MAP1LC3B-II accumulation, an event that was enhanced in the presence of E64d + pepstatin A; Fig. 1A lower panel, Fig. 1B and Fig. S1C) whereas stimulation of the autophagic flux by THC occurred at longer times and was sustained for several hours (Fig. 1A lower panel, Fig. 1B and Fig. S1C).

Previous reports by our group show that the stimulation of sphingolipid biosynthesis by THC is involved in the induction of autophagy-mediated cancer cell death.^20,21,24,26,27^ In agreement with these observations, we found here that THC upregulates mRNA levels of different enzymes involved in sphingolipid synthesis *de novo*, an effect that was not observed when cells were exposed to EBSS (Fig. 1C). Likewise, pharmacological blockade (by using ISP-1) of SPT (serine palmitoyltransferase), the enzyme that catalyzes the first step of sphingolipid biosynthesis, prevented THC-, but not nutrient deprivation-induced autophagy (Fig. 1D). In addition, we confirmed that, in agreement with previous observations,^22,24,27^ incubation with ISP-1 inhibited THC-evoked cell death (Fig. S1D). Collectively, these results suggest that a general increase in *de novo*-synthesized sphingolipids might be a differential factor in the activation of cytotoxic autophagy by THC.

### THC, but not nutrient deprivation, enhances sphingolipid synthesis *de novo* and dihydroceramide accumulation

**Figure 2.**
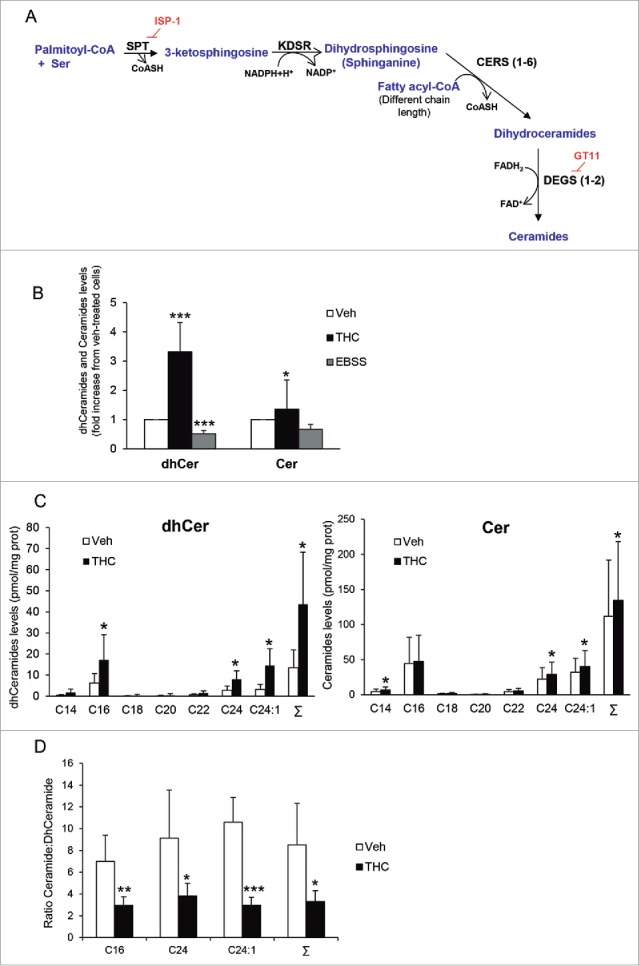
THC, but not nutrient deprivation, stimulates sphingolipid synthesis de novo, enhances dihydroceramide levels and modifies the ceramide:dihydroceramide ratio in the microsomal fraction of U87MG cells. (A) Scheme depicting the pathway of sphingolipid synthesis *de novo*. SPT (serine palmitoyltransferase) catalyzes the condensation of serine and palmitoyl-CoA to produce 3-ketosphinganine. KDSR (3-ketodihydrosphingosine reductase) catalyzes the reduction of 3-ketosphinganine to dihydrosphingosine (sphinganine). The next reaction is catalyzed by CERS1 to CERS6 (each isoform of this enzyme has selectivity for fatty acyl-CoAs with different chain length). CERSs convert dihydrosphingosine into the different molecular species of dihydroceramides, which are subsequently transformed into ceramides by the insertion of a 4, 5-trans double bond catalyzed by the enzymes DEGS1 and DEGS2. ISP-1 and GT11 are pharmacological inhibitors of SPT and DEGS, respectively (B) Effect of THC treatment (6 µM, 6 h) and nutrient deprivation (EBSS; 6 h) on the levels of total ceramides and dihydroceramides found in the microsomal fraction of U87MG cells. Data are expressed as the mean fold change in the levels of total dihydroceramides and total ceramides ± s.d. relative to vehicle-treated cells (n = 4; ***, *P*< 0.001 and *, *P* < 0.05 from vehicle-treated cells). (C) Effect of THC treatment (6 µM, 6 h) on the levels of the different molecular species of dihydroceramides (left panel) and ceramides (right panel) found in the microsomal fraction of U87MG cells. Data are expressed in pmol of sphingolipid per mg of protein (mean ± s.d; n = 5; *, *P* < 0.05 from vehicle-treated cells). Σ indicates the total content in ceramide or dihydroceramide (expressed as the sum of the individual molecular species of ceramide or dihydroceramide detected in these analyses) (D) Effect of THC treatment (6 µM; 6 h) on the ceramide:dihydroceramide ratio ± s.d. found in the microsomal fraction of U87MG cells. (n = 5; ***, *P* < 0.001; **, *P* < 0.01; and *, *P* < 0.05 from vehicle-treated cells). Note that THC treatment produces an increase in the levels of different species of dihydroceramides, which leads to a change in the ratio of both types of sphingolipids in the microsomal fraction of U87MG cells.

The initial steps of sphingolipid biosynthesis occur at the ER,^28^ where ceramides are synthesized (Fig. 2A). Therefore, as a first approach to investigate the effect of THC and nutrient deprivation on sphingolipid metabolism, we analyzed the sphingolipid composition of the microsomal fraction of U87MG cells subjected to either stimulus. As shown in Fig. 2B, THC—but not incubation with EBSS—increased ceramide levels in the microsomal fraction of U87MG cells. We also found that THC but not EBSS enhanced the levels of dihydroceramides to a higher extent than those of ceramides (Fig. 2B and Fig. 2C). DEGS1/dihydroceramide desaturase (delta[4]-desaturase, sphingolipid 1) catalyzes the insertion of a 4,5-*trans* double bond in the sphingoid backbone of dihydroceramides to generate ceramides (see Fig. 2A).^28^ Specifically, treatment with THC produced a 2.8-, 2.9- and 4.5-fold increase in the levels of C16, C24 and C24:1 dihydroceramides, respectively, and a 1.3- and 1.2-fold increase in the levels of C24 and C24:1 ceramides, respectively (Fig. S2). It should be noted that ceramide levels were 6- to 10-fold higher than those of dihydroceramides in vehicle-treated cells (Fig. 2C and Table S1). Thus, the observed increase in dihydroceramides levels triggered by THC led to a striking modification of the ceramide:dihydroceramide ratio in the microsomal fraction of U87MG cells (Fig. 2D).

### THC, but not nutrient deprivation, inhibits sphingolipid transport from the ER to the Golgi

**Figure 3.**
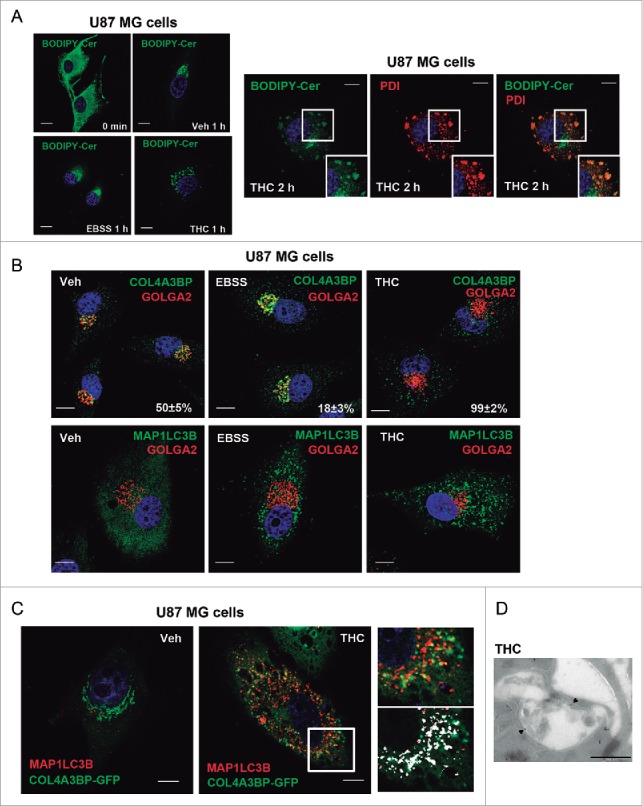
THC, but not nutrient deprivation, inhibits sphingolipid transport from the ER to the Golgi. (A) Effect of THC (4 µM) and EBSS on BODIPY C5 ceramide (BODIPY-Cer) distribution of U87MG cells (cells were incubated at 4°C in the presence of BODIPY C5 ceramide, treated with THC or EBSS and incubated at 37°C for the indicated time) (n = 4). Note that THC, but not nutrient deprivation, produces an accumulation of BODIPY C5 ceramide in vesicles. Right panel: Effect of THC (4 µM, 2 h) on the colocalization of BODIPY C5 ceramide and the ER marker PDIA (protein disulfide isomerase family A member) (n = 4). Bar: 20 μm. (B) Effect of THC (4 µM, 18 h) and EBSS on the subcellular distribution of the ceramide transporter protein COL4A3BP/CERT (n = 4). A representative experiment is shown. Values in the lower right corner of each photomicrograph correspond to the percentage of cells ± s.d. exhibiting a vesicular distribution of COL4A3BP. Note that COL4A3BP colocalizes with the Golgi marker GOLGA2/GM130 in EBSS but not in THC-treated cells (upper panels) and that both EBSS and THC trigger autophagy (as determined by the presence of MAP1LC3B-positive dots; lower panels) under these experimental conditions. Bar: 20 μm. (C) Effect of THC (4 µM, 18 h) on the subcellular distribution COL4A3BP-GFP in MAP1LC3B-positive vesicles. Bar: 20 μm. Right panels correspond to a higher magnification image of the cell region marked with a white square in the middle panel. The bottom right panel shows the colocalization of COL4A3BP-GFP and MAP1LC3B (white spots) in that specific cell region. (D) Immunodectection of COL4A3BP by electron microscopy. Note the presence of COL4A3BP (black spots, marked with black triangles) in double-membrane vesicles present in THC-treated cells (right panel). Bar: 500 nm. Representative electron microscopy images of COL4A3BP immunodetection in vehicle (Veh)- and THC-treated cells are shown in Figure S3C.

Once synthesized in the ER, ceramides can be delivered via vesicular transport or through the ceramide transporter protein COL4A3BP/CERT (collagen, type IV, α 3 [Goodpasture antigen] binding protein)^29^ to the Golgi apparatus, where the synthesis of sphingomyelin and complex glycosphingolipids takes place.^28^ One way to approach the analysis of this process is to follow the subcellular distribution of fluorescent dye-conjugated ceramides, for example BODIPY C5 ceramide. Thus, when added to cells, BODIPY C5 ceramide is endocytosed and rapidly transported to the Golgi. We therefore monitored BODIPY C5 ceramide distribution to analyze the effect of THC on ceramide transport from the ER to the Golgi. As shown in Fig. 3A, BODIPY C5 ceramide was located in perinuclear structures resembling the Golgi apparatus in U87MG cells treated with vehicle or subjected to nutrient deprivation. In contrast, THC treatment induced a particulate distribution of BODIPY C5 ceramide (Fig. 3A). Moreover, this probe colocalized with the ER marker PDIA (protein disulfide isomerase associated) (Fig. 3A, right panel) and showed a striking decrease in the colocalization with the Golgi marker TGOLN2/TGN46 (trans-golgi network protein 2) as compared with vehicle-treated cells (Fig. S3A), suggesting that THC, but not nutrient deprivation, affects the intracellular trafficking of sphingolipids by favoring the accumulation of sphingolipids in the ER.

In agreement with this idea, analysis of COL4A3BP distribution revealed that this protein was located in the Golgi apparatus of vehicle- or EBSS-treated cells, whereas it exhibited a particulate distribution upon challenge with THC (Fig. 3B). We also found that treatment with THC enhanced COL4A3BP phosphorylation (Fig. S3B), an event that promotes a conformational change in this protein that inhibits its ability to transport ceramide from the ER to the Golgi.^30-32^ Surprisingly, immunostaining analyses revealed that THC triggered the colocalization of COL4A3BP with MAP1LC3B-positive dots (Fig. 3C). Moreover, electron microscopy analysis of cells that had been treated with THC showed that COL4A3BP was present in the membrane of vesicles with the morphology of phagophores/autophagosomes (Fig. 3D, and Fig. S3C). Furthermore, the colocalization of COL4A3BP and MAP1LC3B in response to THC was strongly reduced when Ser132—the residue that is primarily phosphorylated to promote COL4A3BP inactivation—was mutated to Ala (Fig. S3D and Fig. S3E), suggesting that THC promotes the phosphorylation and inactivation of COL4A3BP and its localization in phagophores and/or autophagosomes.

To investigate whether COL4A3BP associates with omegasomes we analyzed COL4A3BP localization in ATG5-deficient cells. Note that ATG5 deficiency impairs autophagosome elongation, but not the formation of omegasomes^33^ that can still be detected in these cells when subjected to autophagic stimuli. In line with this idea, treatment with THC promoted the recruitment of COL4A3BP to ring-shaped structures resembling those previously described as characteristic of omegasomes (Fig. S4A). Moreover, THC promoted the colocalization of COL4A3BP with ZFYVE1/DFCP1 (zinc finger, FYVE domain containing 1) (Fig. S4B) and WIPI1 (WD repeat domain, phosphoinositide interacting 1) (Fig. S4C), 2 proteins located in these structures.^3,33^ Taken together, these observations support the conclusion that THC, but not nutrient deprivation, enhances sphingolipid biosynthesis and inhibits the transport of sphingolipids from the ER to the Golgi.

### THC, but not nutrient deprivation, modifies the sphingolipid composition of autophagosomes

**Figure 4.**
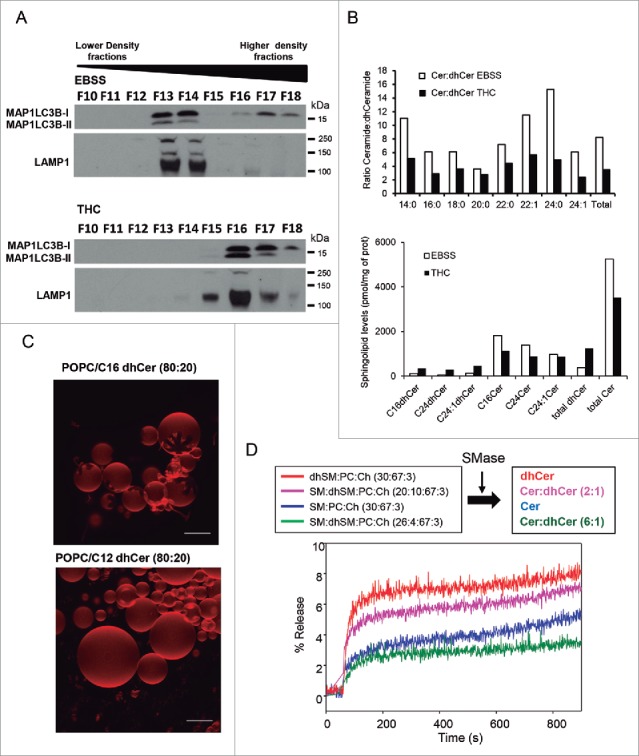
THC, but not nutrient deprivation, decreases the ceramide:dihydroceramide ratio in an autophagosome-enriched fraction. (A) Characterization of the presence of the autophagy marker MAP1LC3B-II and the lysosomal marker LAMP1 in fractions obtained from U87MG cells incubated for 6 h with EBSS or THC (6 µM) and subjected to subcellular fractionation in an OptiPrep® gradient. Note that MAP1LC3B-II appears in fractions of higher density in samples derived from THC-treated cells than in those derived from cells incubated with EBSS, (n = 2). (B) Analysis of the molecular species of ceramides and dihydroceramides present in the MAP1LC3B-II-enriched fraction (derived from cells treated with THC or incubated with EBSS) shown in (A). Data correspond to the ceramide:dihydroceramide ratio (upper panel) and the amount of each sphingolipid species (lower panel) in one representative experiment (n = 2). (C) Generation of ceramide rigid domains in C16 dihydroceramide-containing GUVs. Upper panel: Rigid, dihydroceramide-enriched domains (flower-like dark areas) in bilayers containing 80 mol % *sn*-1-palmitoyl-2-oleoyl phosphatidylcholine (POPC, a fluid phospholipid) and 20 mol % C16 dihydroceramide. Lower panel: a control experiment with a C12 dihydroceramide that does not give rise to domains under these conditions. Bars: 10 µm. (D) Release of vesicular aqueous contents induced by ceramides. Effect of the different proportions of C16 ceramide:C16 dihydroceramide generated by the action of sphingomyelinase in LUVs composed of the following: dhSM:PC:Ch (30:67:3; red line); SM:dhSM:PC:Ch (20:10:67:3; magenta line); SM:PC:Ch (30:67:3; blue line); and SM:dhSM:PC:Ch (26:4:67:3; green line). A representative example of 3 closely similar experiments is shown. SM, sphingomyelin; dhSM, dihydrosphingomyelin; PC, phosphatidylcholine; Ch, cholesterol.

Next we asked whether the changes induced by THC on the ER sphingolipid composition could lead to changes in the sphingolipid composition of autophagosomes. To investigate this possibility, we performed subcellular fractionation experiments to analyze the characteristics of the autophagosomal fraction of U87MG cells treated with THC or subjected to nutrient deprivation. As shown in Fig. 4A and Fig. S5A, the autophagosome-enriched fraction derived from cells that had been treated with THC exhibited a higher density (corresponding to a higher fraction) than that from cells treated with EBSS. Moreover, analysis of the sphingolipid composition of these fractions revealed that dihydroceramide levels were higher (and therefore the ceramide:dihydroceramide ratios were lower) in the autophagosome-enriched fraction from THC-treated cells than in that obtained from cells exposed to EBSS (Fig. 4B).

### Dihydroceramides destabilize biological membranes

To investigate the potential relevance of the changes observed in the sphingolipid composition of autophagosomes and autolysosomes, and specifically of the increased dihydroceramides levels in THC-treated cells, we undertook a series of experiments to analyze the role of these lipids in model vesicles. As THC produced a larger increase in the levels of C16 dihydroceramide (C16-dhCer) than in those of other dihydroceramides (Fig. 2C and Fig. S8A), we selected this molecule to carry out these studies. Experiments performed with giant unilamellar vesicles (GUVs) indicated that C16-dhCer gives rise to flower-shaped, rigid domains (characteristic of inhomogeneous membrane regions)^34^ in these vesicles (Fig. 4C). A control experiment with a shorter chain (C12) dihydroceramide failed to cause lateral domain formation, supporting the notion that (as occurs with ceramides)^34^ under these conditions only long-chain dihydroceramides give rise to rigid domains.

Likewise, calorimetric phase transition experiments showed that C16-dhCer (prepared in a mixture with egg phosphatidylcholine) exhibited a more complex transition, (extending over higher temperatures) than C16 ceramide (C16-Cer) (Fig. S5B); i.e. the membrane rigidifying effect of C16-dhCer is higher than that of C16-Cer. These observations suggest that an enhanced proportion of dihydroceramide facilitates the formation of rigid domains in biological membranes. We therefore analyzed whether these dihydroceramide-enriched domains can contribute to membrane destabilization. To this aim we used large unilamellar vesicles (LUVs) loaded with a water-soluble fluorescent dye. Changes in membrane stability of these vesicles can be determined by measuring the release of the vesicle's aqueous contents. Thus, addition of bacterial sphingomyelinase to LUVs containing different proportions of C16 sphingomyelin and C16 dihydrosphingomyelin led to the formation of ceramide and/or dihydroceramide in the membrane of these vesicles, allowing for the analysis of the effect of acute increases in the level of ceramide and/or dihydroceramide on membrane stability. As shown in Fig. S5C, the release of the vesicle's aqueous contents induced by dihydroceramide was larger and faster than that induced by ceramide.

Next, we prepared vesicles with lipid compositions that mimicked that of the microsomal and autophagosome-enriched fraction of cells treated with THC or EBSS (containing sphingomyelin and dihydrosphingomyelin in the same proportion as those of ceramides and dihydroceramides after treatment with THC or EBSS). Addition of sphingomyelinase to these membranes showed that a higher proportion of dihydroceramides resulted in a more rapid and extensive release of aqueous contents from these vesicles (Fig. 4D). Taken together, these observations support the notion that a decrease in the ceramide:dihydroceramide ratio (similar to that induced by THC in the microsomal and autophagosome-enriched fraction of live cells) leads to the formation of specific membrane domains and to a subsequent destabilization (increased permeability) of the membrane.

### THC promotes sphingolipid- and autophagy-dependent lysosomal membrane permeabilization

**Figure 5.**
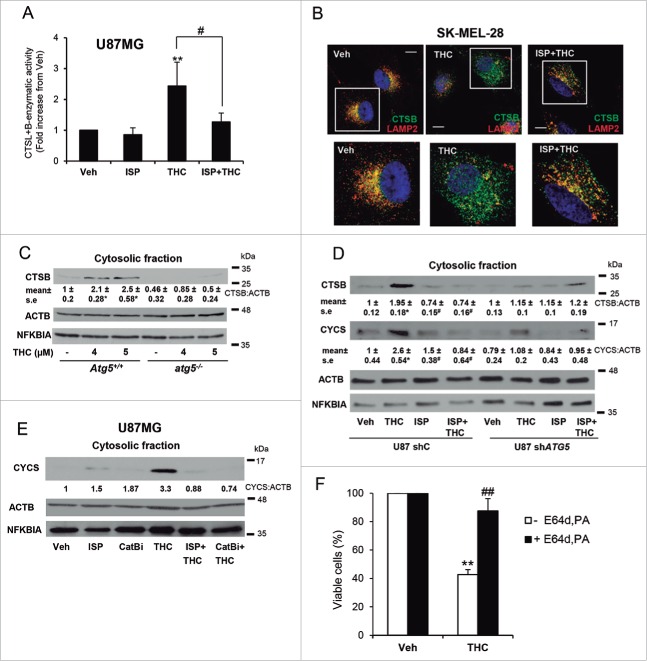
THC promotes lysosomal membrane permeabilization in a sphingolipid- and autophagy-dependent manner. (A) Effect of THC (4 µM) and ISP-1 (5 μM) on CTSB (cathepsin B) + CTSL (cathepsin L) cysteine protease activity in the cytosolic fraction of U87MG cells (16 h). Data are expressed as the mean fold increase in cytosolic CTSB + CTSL cysteine protease activity ± s.d. relative to vehicle-treated cells (n = 4; **, *P*< 0.01 from vehicle-treated cells; and ^#^, *P* < 0.05 from THC-treated cells). (B) Effect of THC (4 µM) and ISP-1 (5 µM) on CTSB and LAMP2 (lysosomal-associated membrane protein 2) subcellular distribution (as determined by immunofluorescence) of SK-MEL28 metastatic melanoma cells (n = 3). Bar: 20 μm. Bottom panels correspond to higher magnification images of the cells marked with white squares in the upper panels. Single and merged channels for these microphotographs are shown in Fig. S6A. (C) Effect of THC (18 h) on CTSB distribution in the cytosolic fraction of *Atg5*^+/+^ or *atg5*^−/−^ (autophagy-deficient) HRASV12/T-large-transformed MEFs (n = 3). Western blots of a representative experiment are shown. NFKBIA/IκBα (NFKB inhibitor α) is included as a control for the presence of cytosolic proteins in the cytosolic fraction. Values in the bottom of the western blots correspond to the mean fold change in the mature CTSB to ACTB/β-actin ratio ± s.e. relative to vehicle-treated *Atg5*^+/+^ cells (n = 3; **, *P* < 0.05 from vehicle-treated cells). Analysis of CTSB distribution in the membrane fraction is shown in Fig. S6B. (D) Effect of THC (4 µM, 16 h) on CTSB and CYCS (cytochrome c, somatic) distribution in the cytosolic fraction of shC and sh*ATG5* U87MG cells (n = 3; a representative western blot is shown). NFKBIA is included as a control of the presence of cytosolic proteins in the cytosolic fraction. Values in the bottom of the western blots correspond to the fold change in the mature CTSB to ACTB ratio ± s.e. and in the CYCS to ACTB ratio ± s.e., respectively, relative to shC U87MG vehicle-treated cells (n = 3; *, *P* < 0.05 from vehicle-treated cells; and ^#^, *P* < 0.05 from THC-treated cells). Analysis of CTSB and CYCS distribution in the membrane fraction is shown in Fig. S6C. (E) Effect of THC (4 µM), ISP-1 (5 µM) and CTSB inhibitor (10 µM) on CYCS distribution in the cytosolic fraction of U87MG cells (n = 2; a representative western blot is shown). NFKBIA is included as a control of the presence of cytosolic proteins in the cytosolic fraction. Values in the bottom of the western blot correspond to the fold change in the CYCS to ACTB (actin, β) ratio relative to U87MG vehicle-treated cells. Analysis of CYCS distribution in the membrane fraction is shown in Fig. S6D. (F) Effect of THC (5 μM) and of the cysteine protease inhibitor E64d (10 μM) and the aspartic protease inhibitor pepstatin A (PA; 10 μg/ml) on the number of U87MG cells (as estimated by the MTT test, 18 h) (n = 4; **, *P* < 0.01; ^##^, *P* < 0.01).

Lysosomal membrane permeabilization (LMP) produces cell death as a consequence of the release of lysosomal proteases to the cytoplasm.^35,36^ Therefore, considering the above-described membrane permeabilizing effect of dihydroceramide, we investigated whether the cell death promoting activity of THC relies on a sphingolipid-dependent induction of LMP. In line with this idea, treatment with THC produced an increase in cytosolic CTSB (cathepsin B) and CTSL (cathepsin L) activity and caused the appearance of CTSB in the cytosol of both U87MG cells and the melanoma cell line SK-MEL-28, these events being prevented by the pharmacological inhibition of sphingolipid synthesis *de novo* (Fig. 5A, Fig. 5B and Fig. S6A). Moreover, we found that THC-induced CTSB release was abrogated in U87MG and SK-MEL-28 cells and in oncogene-transformed MEFs in which autophagy had been genetically inhibited (Fig. 5C, Fig. 5D, Fig. S6B, Fig. S6C, Fig. S7A and Fig. S7B), indicating that autophagy stimulation is required for THC-induced LMP. Collectively these observations suggest that the increase in the dihydroceramide autophagosomal content that triggers THC leads to autolysosomal membrane destabilization, LMP and cathepsin release.

LMP triggers the activation of the mitochondrial apoptotic pathway although it can also lead to necrotic cell death.^36-38^ In agreement with our previous findings showing that autophagy is upstream of apoptosis in the mechanism of cannabinoid-induced cell death,^24^ we found that treatment with THC induced apoptosis and did not lead to a significant increase in necrotic cell death (Fig. S7D). Hence, we next tested whether CYCS (cytochrome c, somatic) release from mitochondria (an event that is closely associated with the activation of the intrinsic apoptotic pathway) was regulated by THC-induced LMP. Supporting this hypothesis, THC treatment promoted mitochondrial CYCS release, an event which was prevented by the pharmacological inhibition of sphingolipid biosynthesis and CTSB activity (Fig. 5D and Fig. 5E, Fig. S6C and Fig. S6D). Likewise, genetic inhibition of autophagy prevented THC-induced CTSB and CYCS release (Fig. 5D and Fig. S6C). Furthermore, pharmacological inhibition of cathepsins prevented THC-induced cell death (Fig. 5F and Fig. S7C). Taken together, these findings show that THC-induced autophagy promotes LMP and the subsequent activation of the mitochondrial apoptotic pathway in a sphingolipid biosynthesis- and autophagy-dependent manner.

### Pharmacological manipulation of the dihydroceramide content activates autophagy-mediated cancer cell death and inhibits tumor growth *in vivo*

**Figure 6.**
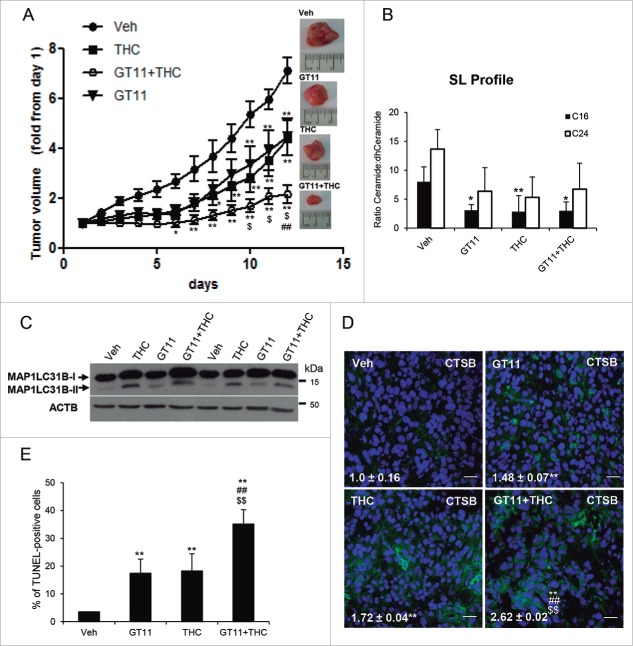
Pharmacological manipulation of the dihydroceramide content of cancer cells activates autophagy-mediated cell death in vivo and inhibits the growth of U87MG cell-derived xenografts. (A) Effect of THC (15 mg/kg; peritumoral administration), GT11 (7.5 mg/kg, peritumoral administration) or THC and GT11 on the growth of tumors generated by subcutaneous injection of U87MG cells. Data are expressed as mean fold increase ± SEM relative to d 1 (n = 6 for each experimental condition; **, *P*< 0.01 or *, *P* < 0.05 from vehicle-treated tumors; ^##^, *P* < 0.01 from THC-treated tumors and ^$^, *P* < 0.05 from GT11-treated tumors). (B) Effect of THC (15 mg/kg), GT11 (7.5 mg/kg) or THC and GT11 on the ceramide:dihydroceramide ratio of tumors generated with U87MG cells. (n = 3; **, *P* < 0.01 or *, *P* < 0.05 from vehicle-treated tumors). (C) Effect of THC (15 mg/kg), GT11 (7,5 mg/kg) or THC and GT11 on autophagy (as determined by MAP1LC3B lipidation). Western blot corresponds to the analysis of 2 different animals/tumors per experimental condition. (D) Effect of THC (15 mg/kg), GT11 (7.5 mg/kg) or THC and GT11 on CTSB immunostaining. Values in the lower left corner correspond to the CTSB-stained area relative to the number of nuclei in each field; these correspond to 10 fields of 3 different tumors for each condition and are expressed as the mean fold change ± s.d. **, *P* < 0.01 from vehicle-treated tumors; ^##^, *P* < 0.01 from GT11-treated tumors and from THC-treated tumors. Representative images from each experimental condition are shown. Bar: 20 μm. (E) Effect of THC (15 mg/kg), GT11 (7.5 mg/kg) or THC and GT11 on apoptosis (as determined by TUNEL). Bars indicate the percentage of TUNEL-positive cells relative to the number of nuclei in each field and correspond to 10 fields of 3 different tumors for each condition and are expressed as the mean fold change ± s.d. **, *P* < 0.01 from vehicle-treated tumors ^##^, *P* < 0.01 from GT11-treated tumors and from THC-treated tumors.

To investigate the in vivo relevance of our observations we analyzed the effect of THC on the growth of U87MG cell-derived subcutaneous tumor xenografts. Treatment with THC reduced tumor growth (Fig. 6A), which correlated with an increase in the levels of C16 dihydroceramide and a decrease in the ratio ceramide:dihydroceramide (Fig. 6B). Likewise, analysis of these samples revealed that treatment with THC enhanced autophagy (as determined by MAP1LC3B lipidation) (Fig. 6C); increased the intensity of CTSB immunostaining (Fig. 6D); and enhanced apoptosis (as determined by TUNEL) (Fig. 6E and Fig. S8F). Taken together, these observations indicate that treatment with THC activates the autophagy-mediated cell death pathway in vivo.

Finally, we questioned whether manipulation of the dihydrosphingolipid content of glioma cells by other means might also facilitate the stimulation of autophagy-mediated cell death. To this aim we analyzed the effect of the DEGS1 pharmacological inhibitor GT11.^39^ As expected, GT11 enhanced dihydroceramide levels and decreased total ceramide levels of U87MG cells (Fig. S8A). Likewise, incubation with this inhibitor induced autophagy (Fig. S8B), CTSB + CTSL release (Fig. S8C) and cell death (Fig. S8D), and enhanced the effect of submaximal doses of THC (Fig. S8B, Fig. S8D and Fig. S8E). Moreover, GT11 decreased the growth of U87MG cell-derived subcutaneous tumor xenografts to a similar extent than THC and enhanced the anticancer activity of this cannabinoid (Fig. 6A). Analysis of samples derived from these tumors showed that treatment with GT11 decreased the C16-ceramide:C16-dihydroceramide ratio (Fig. 6B), and enhanced autophagy (Fig. 6C), CTSB staining and apoptosis (Fig. 6E) to a similar extent as THC. Furthermore, the combined administration of THC and GT11 enhanced CTSB staining and apoptosis in these tumors (Fig. 6D, Fig. 6E and Fig. S8F). Taken together, these observations support the notion that pharmacological manipulation of dihydroceramide levels could be used as a strategy to stimulate autophagy-mediated cancer cell death in vivo.

### Discussion

To investigate the molecular mechanisms that determine the outcome (protective or cytotoxic) of autophagy activation, in this work we compared the effect of 2 autophagic stimuli, namely nutrient deprivation and THC treatment, which activate cytoprotective or cytotoxic autophagy in cancer cells, respectively. Our findings show that THC, but not nutrient deprivation, triggers changes in the sphingolipid composition of the ER (especially an increase in the dihydroceramide:ceramide proportion) and that these changes play a crucial role in the stimulation of autophagy-mediated cancer cell death by THC. Specifically, data support the hypothesis that the THC-promoted modification of the sphingolipid composition of cancer cells is based on its ability to (i) stimulate sphingolipid synthesis *de novo* (via enhanced expression of several genes encoding enzymes of this pathway) and (ii) inhibit the transport of sphingolipids from the ER to the Golgi (at least in part via inhibition of the ceramide transporter protein COL4A3BP). In addition, since THC increases the levels of dihydroceramides to a higher extent than it does with those of ceramides, this agent might also trigger a partial inhibition of DEGS1 (the enzyme that catalyzes the conversion of dihydroceramides into ceramides).^28^ The precise regulatory mechanisms by which binding of THC to cannabinoid receptors triggers these changes in the sphingolipid metabolism of cancer cells have not been clarified as yet and are currently under investigation in our laboratories.

In agreement with the notion that autophagosomal membranes are derived, at least in part and under many cellular settings, from the ER,^5,33^ and that the enzymes involved in the synthesis of ceramides are located in this organelle, our data also indicate that changes induced by THC in the sphingolipid composition of the ER are transmitted to the autophagosomes during the process that gives origin to the phagophore/omegasome, and, in turn, to the autolysosome. Local changes in the concentration of different species of sphingolipids (and specifically of ceramides) produce membrane permeabilization through the formation of rigid structures in biological membranes.^40,41^ Data presented here now show that an increase in the proportion of dihydroceramides strongly enhances this effect. Moreover, results obtained using model vesicles reveal that a local increase in the dihydroceramide:ceramide ratio (similar to that induced by THC in the microsomal and autophagosome-enriched fraction of U87MG cells) leads to the formation of specific membrane domains and to increased permeability of biological membranes. It has been recently shown that manipulation of the activity of SMPD1 (sphingomyelinase phosphodiesterase 1, acid lysosomal; a hydrolytic enzyme located primarily in the lysosomes) leads to LMP and stimulation of cancer cell death,^36,42^ suggesting that changes in the sphingolipid composition of lysosomes can affect the stability of this organelle. Findings presented here now show that autophagy is required for THC-induced LMP and support the idea that the fusion of dihydroceramide-enriched autophagosomes with lysosomes leads in turn to a local increase in the proportion of dihydroceramides in specific subdomains of autolysosomes and lysosomes, thereby leading to membrane destabilization, LMP and the subsequent release of cathepsins into the cytoplasm of cancer cells. Of note, ATG7 has been reported to modulate lysosomal photodamage, through a mechanism that is unrelated to autophagy.^43^ In our study we found that both ATG5- and ATG7-deficient cells were resistant to THC-induced LMP. However, *atg7*^−/−^ cells exhibited an enhanced sensitivity to lysosomal photodamage-induced LMP (data not shown). These observations are in line with the notion that autophagy is required for THC-induced LMP and suggest that lack of ATG7 might affect lysosomal stability in response to agents acting directly at this organelle.

Our findings also show that THC-induced autophagy-mediated LMP leads to cell death via stimulation of the mitochondrial apoptotic pathway rather than necrotic cell death. It is worth noting that ceramides had been previously implicated in autophagy-associated cell death via induction of lethal mitophagy.^44^ However, we did not find a significant increase in mitophagy upon treatment with THC (data not shown) indicating that this mechanism is not responsible for the stimulation of autophagy-mediated cell death in response to treatment with this cannabinoid. These findings are in agreement with previous results from our laboratory showing that autophagy is upstream of apoptosis in the mechanism of cannabinoid-induced glioma cell death^24^ and with the notion that LMP can activate apoptosis.^36,37^

**Figure 7.**
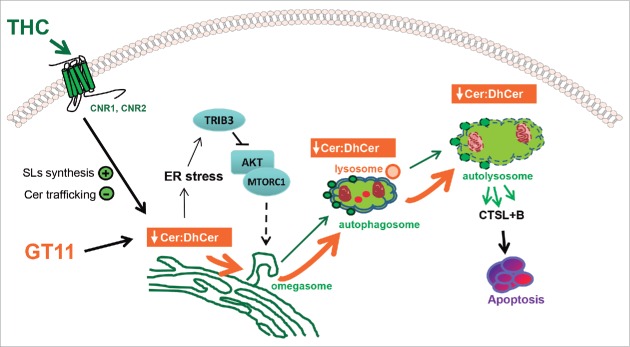
Proposed model of the mechanism by which the intracellular increase of dihydroceramide triggered by THC or by the DEGS inhibitor GT11 promotes glioma cell death. THC binding to CNR1 (cannabinoid receptor 1 [brain]) and CNR2 (cannabinoid receptor 2) stimulates *de novo* synthesis of ceramide and inhibits the transport of ceramide from the ER to the Golgi inducing a modification on the ER sphingolipid composition. This event triggers: (i) the induction of an ER stress response that leads to a TRIB3-dependent inhibition of the AKT-MTORC1 axis and the subsequent induction of autophagy and (ii) a modification of the ceramide to dihydroceramide (Cer:dhCer) ratio in the ER. The DEGS1 inhibitor GT11 produces a similar decrease on the ratio Cer:dhCer. The alteration in the Cer:dhCer ratio triggered by THC or GT11 is transmitted to autophagosomes and autolysosomes, thus modifying the permeability of the membranes, facilitating LMP, cathepsin release and the subsequent activation of apoptosis and cell death.

Different sphingolipids, and specifically ceramides, dihydroceramides and sphingosine 1-phosphate, have been proposed to regulate autophagy in cancer cells primarily by acting as upstream triggers of the signaling pathways that regulate this cellular process.^45-51^ Likewise, previous observations from our laboratory have shown that the stimulation of sphingolipid synthesis *de novo* that triggers THC in glioma and other types of cancer cells elicits an ER stress-related pathway that leads to a TRIB3-dependent inhibition of the AKT-MTORC1 axis and the subsequent activation of autophagy.^24,25^ Findings in the present study now support the notion that the alteration of the sphingolipid metabolism that triggers THC (in addition to activating autophagy via the abovementioned signaling pathway) leads to modified sphingolipid content of the ER, autophagosomes and autolysosomes, and that the latter event plays a crucial role in determining the cell death-promoting fate of autophagy stimulation by cannabinoids (Fig. 7). In any case, further research should clarify whether similar differences in the sphingolipid composition of these organelles may play a role in determining the final outcome of the stimulation of the autophagic process in response to other stimuli.

Of potential relevance in this context, it has been shown that the selective targeting of mitochondria by MAP1LC3B-II-containing phagophores occurs through direct interaction between ceramide and MAP1LC3B-II.^44^ The globular domain of MAP1LC3B was found to be structurally similar to the ceramide-binding domain of COL4A3BP^44^ (which can also bind C16-dihydroceramide).^52^ It is therefore tempting to speculate that, in addition to regulating membrane stability and the activity of the above-described ER stress-related signaling pathway, local changes in the content and subcellular distribution of C16-dihydroceramide or other dihydrosphingolipids might be able to modulate autophagy via selective binding to MAP1LC3B or other autophagy regulatory proteins.

In this report, we also show that treatment with THC or inhibition of DEGS1 efficiently activates autophagy and apoptosis and inhibits tumor growth in mice. These findings support the idea that the pharmacological manipulation of the sphingolipid content (and specifically of the levels of certain species of dihydroceramides) may be exploited therapeutically to promote the activation of autophagy-mediated LMP and cancer cell death. It is tempting to speculate that this strategy could be useful to enhance the efficacy of certain anticancer therapies, for example by turning protective autophagy (that becomes activated as a mechanism of resistance in response to treatment with certain antineoplastic agents)^12,53^ into a cell death-promoting process.

In summary, findings presented in this report support the concept that the stimulation of autophagy-mediated cancer cell death by THC relies on a modification of the sphingolipid composition of the endoplasmic reticulum of glioma cells that is transmitted to autophagosomes and autolysosomes thereby leading to lysosomal membrane permeabilization, cathepsin release and the subsequent activation of apoptotic cell death. We think that these observations contribute to further support the biological relevance of sphingolipid metabolites in the regulation of autophagy and to emphasize the potential therapeutic implications of modulating the levels of dihydrosphingolipids such as dihydroceramides for the treatment of cancer.

## Materials and methods

### Reagents

The following reagents were used: THC (THC Pharm GmbH, THC-1099), pepstatin A (Enzo Life Sciences, ALX-260-085), E64d (Enzo Life Sciences, BML-PI107), myriocin (ISP-1, Sigma-Aldrich, M1177), sphingomyelinase (_EC_ 3.1.4.12) from *Bacillus cereus* (Sigma-Aldrich, S7651), o-phenanthroline (Sigma-Aldrich, 131377) and CA-074 methyl ester (CTSB/cathepsin B inhibitor; Sigma-Aldrich, C5857). Phosphatidylcholine from egg yolk (PC; Lipid Products, grade 1, 840051P), sphingomyelin, (SM; Avanti Polar Lipids, 860061), C12 ceramide (C12-Cer; Avanti Polar Lipids, 860512), C16 dihydroceramide (C16-dhCer; Avanti Polar Lipids, 860634), palmitoyl-oleoylphosphatidylcholine (POPC; Avanti Polar Lipids, 850457) and cholesterol (Ch; Avanti Polar Lipids, 700000). DEGS1 inhibitor GT11, and dihydrosphingomyelin (dhSM) were synthesized in our laboratories. dhSM was synthesized from egg SM (Avanti Polar Lipids, 860061) and contained 86% C16 dhSM. ANTS (Molecular Probes, Inc., A350), DPX (Molecular Probes, Inc., X1525) and DiIC_18_ (Molecular Probes, D3911). BODIPY FL C5-ceramide complexed to BSA (ThermoFisher Scientific, B22650).

### Cell culture

U87MG (human glioma cell line), A375 and SK-MEL28 cells were obtained from the American Type Culture Collection (Rockville, MD, USA; ATCC® HTB-14™, ATCC® CRL-1619™, ATCC® HTB-72™). T-Large antigen-*Atg5*^+/+^ and *atg5*^−/−^ MEFs were transformed using a retroviral vector expressing a mutated (Gly12Val) and constitutively active form of HRAS (Harvey rat sarcoma virus oncogene) (HRAS^G12V^; HRASV12/T-large-MEFs) as previously described.^54^ Transformed/stably transfected MEFs correspond to a polyclonal mix of at least 20 different selected clones. Cells were cultured in DMEM (Lonza, BE-12-604F) containing 10% fetal bovine serum (FBS; Linus, 91S1800) and penicillin/ streptomycin (5 µg/ml; Lonza, BE17-603E). When required, cells were seeded at a density of 5000–10,000 cells/cm^2^ and transferred to medium containing 0.5% FBS, 18 h before performing the different treatments. For nutrient deprivation experiments, cells were incubated in Earle´s balanced salt solution (EBSS) medium (Lonza, BE10-502F). T-large antigen-immortalized *Atg5*^+/+^, *atg*5^−/−^, *Atg7*^+/+^ and *atg7*^−/−^ MEFs were kindly provided by Noboru Mizushima (The University of Tokyo, Japan)

### Infection with *ATG5* shRNA-human lentiviral particles

A pool of concentrated transduction-ready viral particles containing 3 shRNAs target-specific (or 3 shRNA nontargeted control) constructs (19–25 nucleotide plus hairpin; Santa Cruz Biotechnology, sc-41445-V) was used to stably knock down the expression of *ATG5* in U87MG cells. Briefly, cells were plated in 12-well dishes 24 h prior to viral infection. The day after, when the cells reached 50% confluence, medium was removed and replaced by complete medium with hexadimethrine bromide (Sigma- Aldrich, H9268-5G) at a final concentration of 5 µg/ml. Cells were subsequently infected with control- or *ATG5*-selective shRNA lentiviral particles. The day after, the medium was removed and replaced by complete medium without hexadimethrine bromide. Finally, to select the clones stably expressing the shRNAs, the cells were incubated with puromycin (Gibco, 10296974) at a concentration of 2 to 10 µg/ml. Finally, clones were selected and stable silencing was confirmed by different approaches. At least 20 different selected clones were pooled for each of the cell lines generated.

### Real-time quantitative PCR

RNA was isolated by using Trizol Reagent (Sigma- Aldrich, T9424) following the manufacturer´s instructions and including a DNase digestion step with the RNasefree DNase kit (Qiagen, 79254). cDNA was subsequently obtained using the Transcriptor first strand cDNA synthesis kit (Roche, 04897030001). Real-time quantitative PCR assays were performed using the FastStart Master Mix with Rox (Roche, 04914058001) and probes were obtained from the Universal Probe Library Set (Roche). The following primer sequences and Roche´s probes were used for detecting human *CERS2* transcript variant 1 (Forward 5´-GACGGAGTACACGGAGCAG-3´, Reverse 5´-CGTTCCCACCAGAAGTAATCA- 3′ probe 50), human *CERS5* (Forward 5´-GCCATCCTTGAAAAGGTGTT-3´, Reverse 5´-AATCCAGCTGCTTTGACAGG-3´, probe 19), human *CERS6* (Forward 5´-TCATGATTCAGCTGATGCTCTT-3´, Reverse 5´-CACATTTTCTGAAACTTGGCATA-3´ probe 75), human *DEGS1* (Forward 5´-GGAAGACTTCGAGTGGGTCTAC-3´, Reverse 5´- TTCATCAAGGACTTTATCTCTGGA-3´, probe 28), human *SPTLC1* (Forward 5´-CATTAACTCAGGCGCCGTAC-3´, Reverse 5´-GTTCCACCGTGACCACAAC-3´, probe 52). Amplifications were run in a 7900 HT-Fast Real-Time PCR System (Applied Biosystems; California, USA). Each value was adjusted by using *RNA18S1* levels as reference (Forward 5´-GCTCTAGAATTACCACAGTTATCCAA-3, Reverse 5´-AAATCAGTTATGGTTCCTTTGGTC-3´, probe 55).

### Transfections of expression vectors

Transfections of expression vectors were performed with Lipofectamine 2000 (Invitrogen, 11668019) according to the manufacturer´s instructions. Plasmids pEGFP-COL4A3BP and the mutant pEGFP-COL4A3BP S132A have been previously described.^31^ The plasmid encoding ZFYVE1/DFCP1-MYC and WIPI1-HA were kindly provided by Dr Nicholas Ktistakis (Babraham Institute, Cambridge, UK) and Dr. Sharon A. Tooze (The Francis Crick Institute, London, UK), respectively.

### Cell viability assays

Cell viability was determined by the MTT ((3-[4,5-dimethylthiazol-2-yl]-2,5-diphenyltetrazolium bromide, a yellow tetrazole) (Sigma-Aldrich, M2128) test following the manufacturer´s instructions. Absorbance at 570 nm, which is proportional to the amount of viable cells in the culture, was quantified using a spectrophotometer.

### Cell lysates

Cells were lysed in a buffer containing 50 mM Tris HCl (Roth, 20485000), pH 7.5, 1 mM phenylmethylsulfonyl fluoride, 50 mM NaF, 5 mM sodium pyrophosphate, 1 mM sodium orthovanadate, 0.1% Triton X-100, 1 mg/ml leupeptin, 1 mM EDTA, 1 mM EGTA and 10 mM sodium β-glycerophosphate (Sigma-Aldrich, 329-98-6, S7920, T6379, S6508, L8511, ED, E4378, G6251, T9284, respetively).

### Western blot

Western blot analysis was performed following standard procedures.^55^ Primary antibodies raised against NFKBIA (1:2000; Santa Cruz Biotechnology, sc-371), ACTB, TUBA1A, MAP1LC3B, TGOLN2 (1:5000, 1:5000, 1:3000, 1:1000; Sigma-Aldrich, A5441, T9026, L7543, T7576), LAMP2, EEA1 (1:1000, 1:500; BD Biosciences 555803 and 610457), COL4A3BP (1:1000; Bethyl, A300-669A), CANX (1:500; StressMarq, SPC-108B), GOLGA2 (1:1000; Abcam, ab52649), and LAMP1 (1:1000; Abcam, ab24170), were used. Densitometric analysis was performed with Quantity One software (Bio-Rad; California, USA).

### Lipid extraction

Briefly, 1 to 10 × 10^6^ pelleted U87MG cells were mixed with 0.5 ml methanol (Merck, 1.06018.1000) and 0.25 ml chloroform (Scharlab, CL01981000) and internal standards were added (200 pmol C12-Cer, SM, and GlcCer; Avanti Polar Lipids, 860512, 860583, 860543). Samples were heated at 48°C overnight. The next day, 75 μl 1 M KOH (Panreac, 141515.1211) in methanol were added, followed by 2-h incubation at 37°C. Finally, the mixtures were neutralized with 75 μl 1 M acetic acid (Panreac, 161008.1611), and dried under nitrogen.

### Lipidomics

Lipid extracts were solubilized in 150 μl methanol. The liquid chromatography-mass spectrometer consisted of a Waters Aquity UPLC system connected to a Waters LCT Premier Orthogonal Accelerated Time of Flight Mass Spectrometer (Waters, Millford, MA, USA), operated in positive or negative electrospray ionization mode. Full scan spectra from 50 to 1500 Da were obtained. Mass accuracy and reproducibility were maintained by using an independent reference spray via LockSpray. A 100 mm × 2.1 mm id, 1.7 μm C8 Acquity UPLC BEH (Waters) analytical column was used. The 2 mobile phases were 1 mM ammonium formate (Fluka, 09735) in methanol (phase A) and 2 mM ammonium formate in H_2_O (Fisher Scientific, W6-212) (phase B), both phases with 0.05 mM formic acid (Merck, 1.00264.1000). Two gradients were programmed: gradient I: 0 min, 80% A; 3 min, 90% A; 6 min, 90% A; 15 min, 99% A; 18 min, 99% A; 20 min, 80% A and gradient II: 0 min, 65% A; 2 min, 65% A; 5 min, 90% A; 11 min, 99% A; 12 min, 99% A; 14 min, 65% A. In both cases, the flow rate was 0.3 ml/min. The column was run at 30°C. Quantification was carried out using the ion chromatogram obtained for each compound using 50-mDa windows. The linear dynamic range was determined by injection of standard mixtures. Positive identification of compounds was based on the accurate mass measurement with an error <5 ppm and its LC retention time, compared to that of a standard (± 2%). Sphingolipids were annotated as 〈lipid subclass〉〈total fatty acyl chain length〉:〈total number of unsaturated bonds〉. If the sphingoid base residue was dihydrosphingosine the lipid class contained a 〈DH〉 prefix.

### Confocal laser scanning microscopy

Standard protocols for immunofluorescence microscopy were used. Briefly, cell cultures grown on 12-mm coverslips (Menzel Glässer, P231.2) were washed in phosphate-buffered saline (PBS; 137 mM NaCl, 4.3 mM Na_2_HPO_4_, 1.47 mM KH_2_PO_4_, pH 7.5), fixed with 4% paraformaldehyde (Sigma-Aldrich, P6148) (20 min at room temperature) and permeabilized with 0.5% Triton X-100 (5 min at room temperature). Cells were then incubated with the corresponding primary antibodies diluted in PBS containing 0.1% w/v BSA (Sigma-Aldrich, A6003) for 2 h and washed 3 times with this same buffer. Incubation with the appropriate Alexa Fluor 488- or Alexa Fluor 594-conjugated secondary antibodies (1:1000, Invitrogen, A-11008, A-11005, A-11001, R37117) was performed in the dark at room temperature for 90 min. Cell nuclei were stained with DAPI (Roche, 010236276001; 10 min, room temperature). Finally, coverslips were mounted in Mowiol mounting medium (Calbiochem, 475704) and observed in a Leica TCS SP2 confocal microscope. At least 200 cells per condition were counted in randomly selected fields and the results represent the mean value ± STDEV corresponding to the randomly selected fields of a representative experiment. Primary antibodies were as described above and additionally included anti-HA (16B12 clone; Covance MMS-101P), CTSB (EMD Millipore, IM27L), anti-MYC (9E10 clone; Roche, 11667203001), and PDIA/PDI (Abcam, ab3672). In the double immunostaining with LAMP2 and CTSB antibodies, the mouse-on-mouse blocking reagent (Vector Labs, MKB2213) was used.

### Electron microscopy

U87MG cell were chemically fixed at 4°C with a mixture of 2% paraformaldehyde and 0.1% glutaraldehyde (Sigma-Aldrich, 340855) in PBS buffer. After washing with PBS containing 50 mM glycine (Sigma-Aldrich, G7126), cells were embedded in 12% gelatin (Sigma-Aldrich, G1393) and infused in 2.3 M sucrose ((Roth, 4261.1). Mounted gelatine blocks were frozen in liquid nitrogen. Thin sections were prepared in an ultracryomicrotome (Leica EM Ultracut UC6/FC6, Vienna, Austria). Ultrathin cryosections were collected with 2% methylcellulose in 2.3 M sucrose (Roth, 4261.1). Cryosections were incubated at room temperature on drops of 2% gelatin in PBS for 20 min at 37°C, followed by 50 mM glycine in PBS for 15 min and 10% FBS in PBS for 10 min, and finally 5% FBS in PBS for 5 min. Cryosections were subsequently incubated with anti-COL4A3BP (Bethyl, A300-669A) in 5% FBS in PBS for 30 min. After 3 washes with drops of PBS for 10 min, sections were incubated for 20 min using IgG anti-mouse coupled to 10-nm diameter colloidal gold particles (Electron Microscopy Sciences, 25108) using a (1:200) dilution in 5% FBS in PBS. This was followed by 3 washes with drops of PBS for 10 min, and 2 washes with distilled water. As a control for non specific binding of the colloidal gold-conjugated antibody, the primary antibody was omitted. Preparations were observed in an Electron Microscope Tecnai Spirit (FEI Company, The Netherlands) with a CCD camera SIS Megaview III or in a Jeol J1010 (Jeol, Japan) with a CCD camera SIS Megaview III.

### Isolation of the autophagosomal-enriched fraction

U87MG cells were cultured in 150-mm dishes at 10,000 cells/cm^2^ and starved in EBSS medium or treated with THC during 6 h. Then, cells were harvested with a scraper, collected in tubes and centrifuged at 800 x g, at room temperature for 5 min. The package cellular volume (PCV) of the pellet fractions was measured, and each pellet was suspended in 3 volumes of the PCV in hypotonic buffer, incubated for 20 min at 4°C and centrifuged at 600 x g for 5 min. The new PCV was suspended in 2 volumes of isotonic buffer and homogenized with a Potter Elvejem homogenizer. The microsomal fraction was prepared with 3 sequential centrifugations (1,000 x g, 4°C, 5 min; 12,000 x g, 4°C, 15 min; and 100,000 x g, 4°C, 2 h). Next, the pellet fraction was suspended in 0.8 ml 0.25 M sucrose and 1.4 ml of OptiPrepTM (Progen Biotechnik, 11145429), and was placed at the bottom of Ultra-ClearTM Tubes (14×95 mm; Beckman–Coulter, 82355618). A discontinuous OptiPrepTM gradient was constructed by modification of the method described by Marzella et al.^56^ The layers from the bottom to the top were: 3 ml of 26% OptiPrepTM, 2 ml of 24% OptiPrepTM, 2 ml of 20% OptiPrepTM and 2 ml of 15% OptiPrepTM. After centrifugation at 90,017 x g for 3 h at 4°C in an SW40Ti rotor (Beckman Instruments, Spinco Div., Palo Alto, CA), fractions of 0.5 ml were collected and analyzed.

### Liposome preparation

LUVs of diameters 100–150 nm were prepared by the extrusion method using a LIPEX Liposome Extrusion System (Transferra Nanosciences, Burnaby, Canada) equipped with nuclepore filters of 0.1-μm pore diameter (Whatman, 110605), at 65°C in 10 mM HEPES (Sigma-Aldrich, H7006), 150 mM NaCl, 10 mM CaCl2, 2 mM MgCl2, pH 7. The final lipid concentration was 2 mM. GUVs were prepared following the electroformation method described previously,^57^ using a homemade chamber (Industrias Tecnicas ITC, Bizcaya, Spain) that allows direct visualization under the microscope. Stock solutions of lipids (0.2 mg/ml total lipid containing 0.4 mol DiC_18_) were prepared in chloroform:methanol (2:1, v/v) solution. 3 µL of the appropriate stocks were added onto the surface of platinum electrodes, and solvent traces were removed under vacuum for at least 2 h. The platinum electrodes were covered with 400 ml of 25 mM HEPES, 150 mM NaCl, pH 7.5 buffer previously heated at 65°C, and connected to an electric wave generator (TG330 function generator, Thurlby Thandar Instruments, Huntington, UK) under AC field conditions (1] 500 Hz, 0.08 V for 6 min; 2] 500 Hz, 1.0 V for 20 min; 3] 500 Hz, 3.0 V for 1 h 30 min) at 65°C. Phospholipid concentration was measured in terms of lipid phosphorus.^58^

### Fluorescence microscopy

Giant vesicles were visualized in an inverted confocal fluorescence microscope with a high-efficiency spectral detector (Leica TCS SP5; Leica Microsystems CMS GmbH, Mannheim, Germany). The excitation wavelength was 514 nm, and the fluorescence signal was collected in the 570–610 nm channel. Images were collected and analyzed with the LAS AF software (Leica Microsystems).

### Release of vesicle contents was assayed with the ANTS:DPX fluorescence system

Details on the use of these fluorescent probes, including assay calibration, have been given elsewhere.^59,60^ Leakage was followed in terms of ANTS fluorescence at 37°C in a QuantaMaster™ spectrofluorometer series (Photon Technology International, Birmingham, NJ, USA). Since commercial sphingomyelinase preparations may contain phospholipase C impurities, 2 mM o-phenanthroline (Sigma-Aldrich, 131377) was routinely added in all our enzyme assays. The lipid concentration was 0.3 mM and sphingomyelinase was used at 0.15 units/ml.

### Cathepsin activity measurements

A total of 15,000 cells/well were plated and cultured in 12-well plates 1 d prior to THC treatment. Three hundred μl of extraction buffer (250 mM sucrose, 20 mM HEPES, 10 mM KCl [Sigma- Aldrich, P9333], 1.5 mM MgCl_2_, 1 mM EGTA, 1 mM EDTA, 8 mM DTT [Sigma-Aldrich, D0632], 1 mM Pefabloc® SC [Sigma-Aldrich, 30827-99-7], pH 7.5) with either 13 μg/ml (for the cytosolic fraction) or 200 μg/ml (for total protein) of digitonin (Sigma-Aldrich, D141) was added to the cells. After a 12-min incubation at 4°C, 250 μl of the supernatant fraction was transferred to a microtiter plate. Fifty μl of extract per sample were transferred to black Costar 96-well plates into 50 µl of 2x cathepsin reaction buffer (50 mM sodium acetate, 8 mM EDTA, 8 mM DTT, 1 mM Pefabloc® SC, pH 5.0) containing the zFR-AFC (50 μM; Enzo, ALX-260-129-M005) cathepsin substrate. To measure cysteine cathepsin activity, plates were prewarmed for 5 min at 30°C and light emission (max. 489 nm, cutoff at 475 nm; excitation at 400 nm) was measured on a SpectraMax Gemini fluorescent reader (Molecular Devices, Sunnyvale, CA, USA) every 2 min for 30 min.

### CTSB and CYCS release detection

U87MG cells cultured on P100 plates (TPP, 93100) were lysed in plasma membrane permeabilization buffer (50 ug/ml digitonin, 80 mM KCl in PBS), 16 h after the corresponding treatments, and the presence of CYCS or CTSB in the cytosolic fraction were analyzed as previously described.^61^

### *In vivo* treatments

Tumors derived from U87MG cells were induced in Hsd:AthymicNude-Foxn1nu mice (Envigo RMS-Spain) by subcutaneous injection of 9 × 10^6^ cells in PBS supplemented with 0.1% glucose. Tumors were allowed to grow until an average volume of 250–300 mm^3^ and animals were assigned randomly to the different groups. Treatments were administered with a single peritumoral (local) injection, in 100 μl of PBS supplemented with 5 mg/ml BSA. Tumors were measured with external caliper, and volume was calculated as (4π/3) x (width/2)^2^ x (length/2). All procedures involving animals were performed with the approval of the Complutense University Animal Experimentation Committee according to Spanish and European official regulations.

### Immunomicroscopy of tumor samples

Samples from tumor xenografts were dissected, OCT Tissue-Tek (SAKURA FINETEK, E11K4583) embedded and frozen. Standard protocols for immunofluorescence microscopy were used.

### Tunel

Tumor samples were fixed, blocked and permeabilized and TUNEL was performed as previously described.^22^

### Statistics

Statistical analyses were performed by ANOVA with a post hoc analysis by the Student-Neuman-Keuls test.

## Supplementary Material

KAUP_A_1213927_Supplementary_material.zip

## Abbreviations

ACTB: actin, β
ATG: autophagy related
Cer: ceramide
COL4A3BP/CERT: collagen, type IV, α 3 (Goodpasture antigen) binding protein
CTSB: cathepsin B
CYCS: cytochrome c, somatic
DEGS1: delta(4)-desaturase, sphingolipid 1
dhCer: dihydroceramide
EBSS: Earle's balanced salt solution
ER: endoplasmic reticulum
GUVs: giant unilamellar vesicles
LMP: lysosomal membrane permeabilization
LUV: large unilamellar vesicles
MAP1LC3/LC3: microtubule-associated protein 1 light chain 3
MEFs: mouse embryonic fibroblasts
MTORC1: mechanistic target of rapamycin (serine/threonine kinase) complex 1
PCV: package cellular volume
THC: Δ^9^-tetrahydrocannabinol
TRIB3: tribbles pseudokinase 3
