# Supplementary material for: Dihydroceramide accumulation mediates cytotoxic autophagy of cancer cells via autolysosome destabilization
Source: Autophagy. 2016 Sep 16;12(11):2213–29. doi: 10.1080/15548627.2016.1213927 (PMC5103338; doi:10.1080/15548627.2016.1213927)
Supplement: KAUP_A_1213927_Supplementary_material.zip [file kaup-12-11-1213927-s001.zip › KAUP_A_1213927 Supplementary material.pdf]

Hernández-Tiedra et al. Figure S1

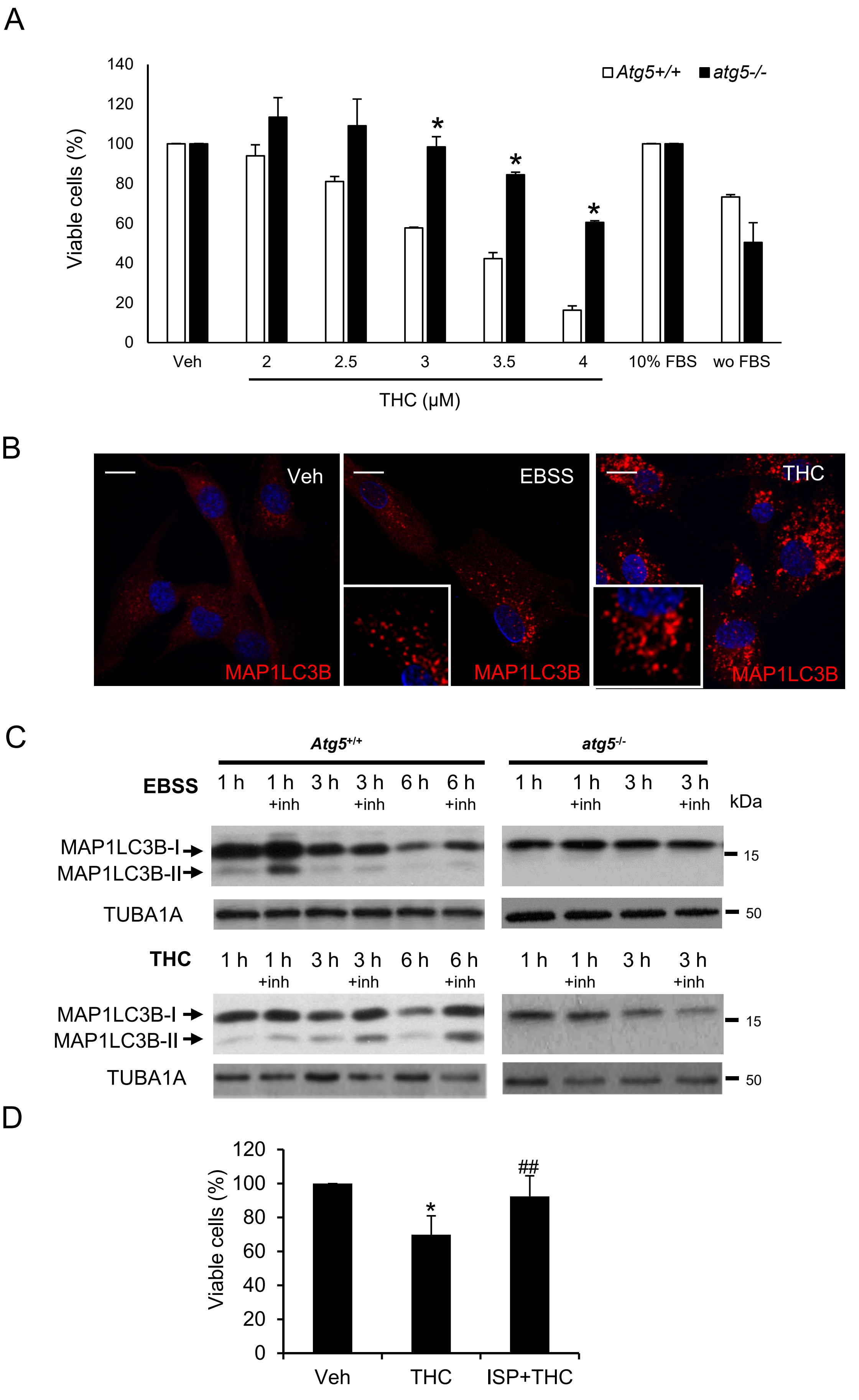

Figure S1. Stimulation of autophagy in response to THC treatment and nutrient deprivation produces a different effect on cancer cell viability. (**A**) Effect of THC (18 h) and nutrient deprivation (18 h) on the number of *Atg5*$^{+/+}$ or *atg5*$^{-/-}$ (autophagy-deficient) HRASV12/T-large-transformed MEFs (as estimated by the MTT assay) (mean ± s.d; n = 4; *, $P < 0.05$ from the corresponding *Atg5*$^{+/+}$ cells). (**B**) Effect of THC (4 µM, 18 h) and EBSS (18 h) on autophagy (as determined by MAP1LC3B immunostaining) of U87MG cells. Note that the morphology of the autophagosomes is different upon exposure to THC and EBSS. Bar: 20 µm. (**C**) Effect of THC (4 µM) and incubation with EBSS (i.e., nutrient deprivation) on the induction of autophagy (as determined by MAP1LC3B lipidation) of *Atg5*$^{+/+}$ or *atg5*$^{-/-}$ (autophagy-deficient) HRASV12/T-large-transformed MEFs (n = 3; a representative experiment is shown). (**D**) Effect of THC (4 µM, 18 h) and myriocin (ISP) on the number of U87MG cells (as estimated by the MTT assay) (mean ± s.d.; n = 3; *, $P < 0.05$ from vehicle (Veh)-treated cells; $^{\#\#}$, $P < 0.01$ from THC-treated cells). No significant differences were found on the number of ISP- and Veh-treated cells (as estimated by the MTT assay).

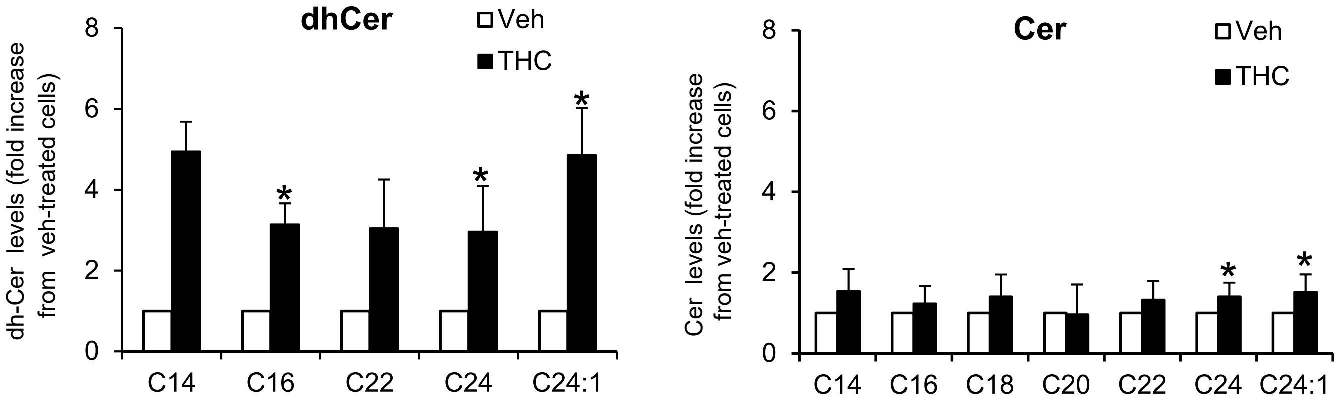

Figure S2. THC modifies the levels of dihydroceramides and ceramides in the microsomal fraction of U87MG cells. Effect of THC treatment (6 μM, 6 h) on the levels of the different molecular species of dihydroceramides (dhCer) and ceramides (Cer) found in the microsomal fraction of U87MG cells. Data correspond to the mean fold increase in the levels of the different molecular species of dihydroceramides (left panel) or ceramides (right panel) ± s.d. relative to vehicle-treated cells (n = 5; *, $P < 0.05$ from vehicle-treated cells).

Hernández-Tiedra et al. Figure S3

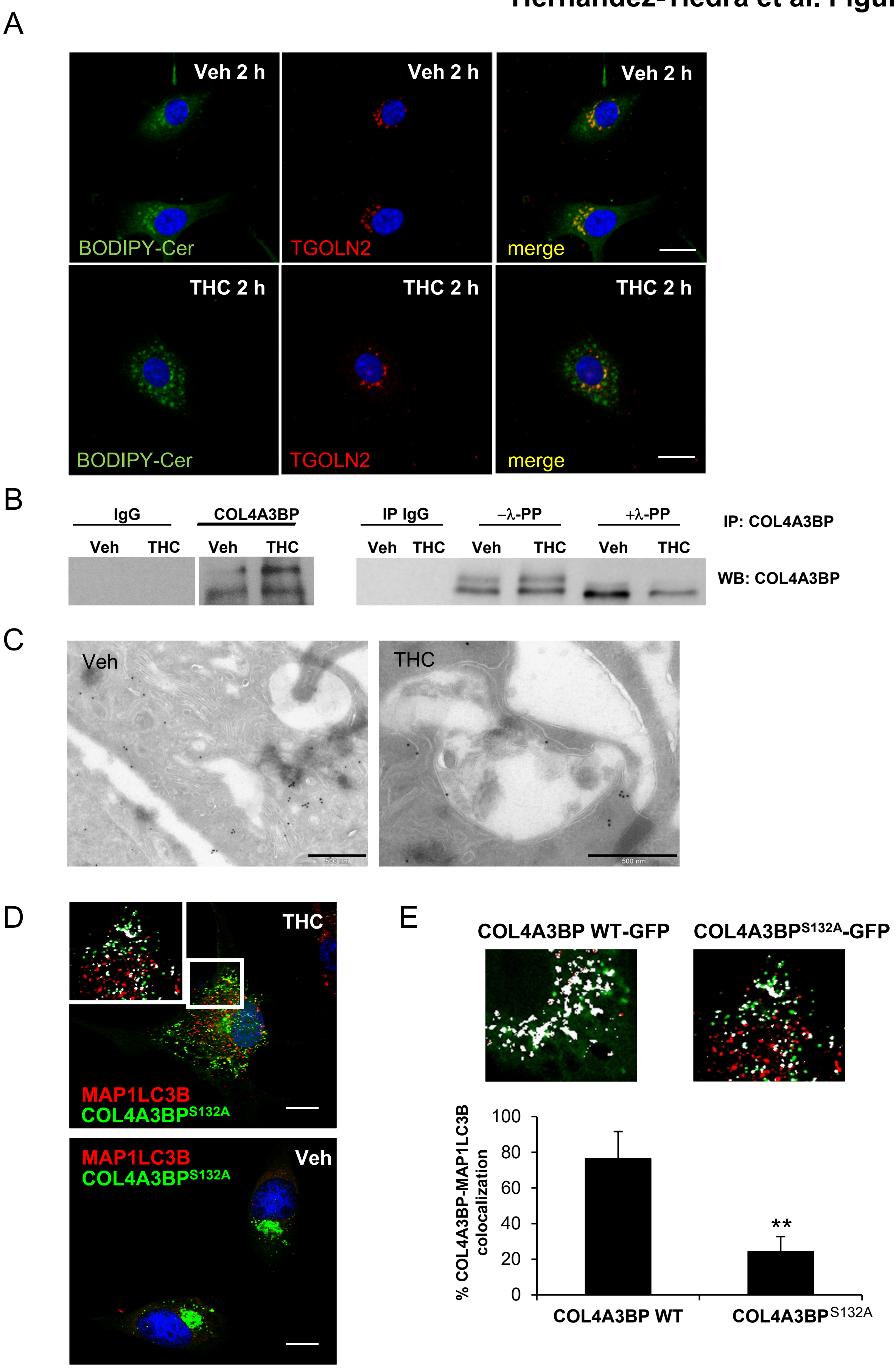

Figure S3. THC modifies sphingolipid trafficking and promotes COL4A3BP phosphorylation. (**A**) Effect of THC (4 µM, 2 h) on BODIPY C5 ceramide (BODIPY-Cer) distribution of U87MG cells (cells were incubated at 4°C in the presence of BODIPY C5 ceramide, treated with THC or vehicle and incubated at 37°C for the indicated time) (n = 3). Note that BODIPY C5 ceramide colocalizes with the Golgi marker TGOLN2/TGN46 (trans-golgi network protein 2) in vehicle-treated, but not in THC-treated, cells. Bar: 20 µm. (**B**) Effect of THC (5 µM, 7 h) on the phosphorylation of COL4A3BP as determined by COL4A3BP immunoprecipitation and subsequent analysis of COL4A3BP band shift in the presence or the absence of the catalytic subunit of PP1 (λ-PP). (**C**) Effect of THC (5 µM, 7 h) on COL4A3BP subcellular distribution as determined by immunogold transmission electron microscopy of U87MG cells. Note that COL4A3BP is associated with the membrane of autophagosome-like structures in THC-treated cells. Bar: 500 nm. (**D**) Effect of THC (5 µM, 7 h) on the colocalization of LC3 and the Ser132Ala mutant form of COL4A3BP-GFP (COL4A3BP^{S132A}) in U87MG cells. The image in the upper right corner corresponds to a higher magnification image of the cell region marked with a white square and shows the colocalization of COL4A3BP-GFP and MAP1LC3B (white spots) in that specific cell region. Note that the S132A mutation abolishes the THC-induced colocalization of COL4A3BP-GFP and MAP1LC3B. Bar: 20 µm. (**E**) Analysis of the colocalization of COL4A3BP wild type-GFP (COL4A3BP WT) and COL4A3BP^{S132A}-GFP (COL4A3BP^{S132A}) and MAP1LC3B in U87MG cells treated with THC (5 µM, 7 h). Images in the upper panels correspond to the cell region marked with a white square in Fig. 3C middle panel (COL4A3BP WT) and to the cell region marked with a white square in Fig. S3D (COL4A3BP^{S132A}). Data in the lower panel correspond to the quantification of the colocalization of COL4A3BP WT or COL4A3BP^{S132A} with MAP1LC3B and are expressed as the percentage of colocalization of MAP1LC3B and COL4A3BP WT or COL4A3BP^{S132A} ± s.d. (n = 3; **, P < 0.01 from COL4A3BP WT-transfected U87MG cells). Bar: 20 µm.

Hernández-Tiedra et al. Figure S4

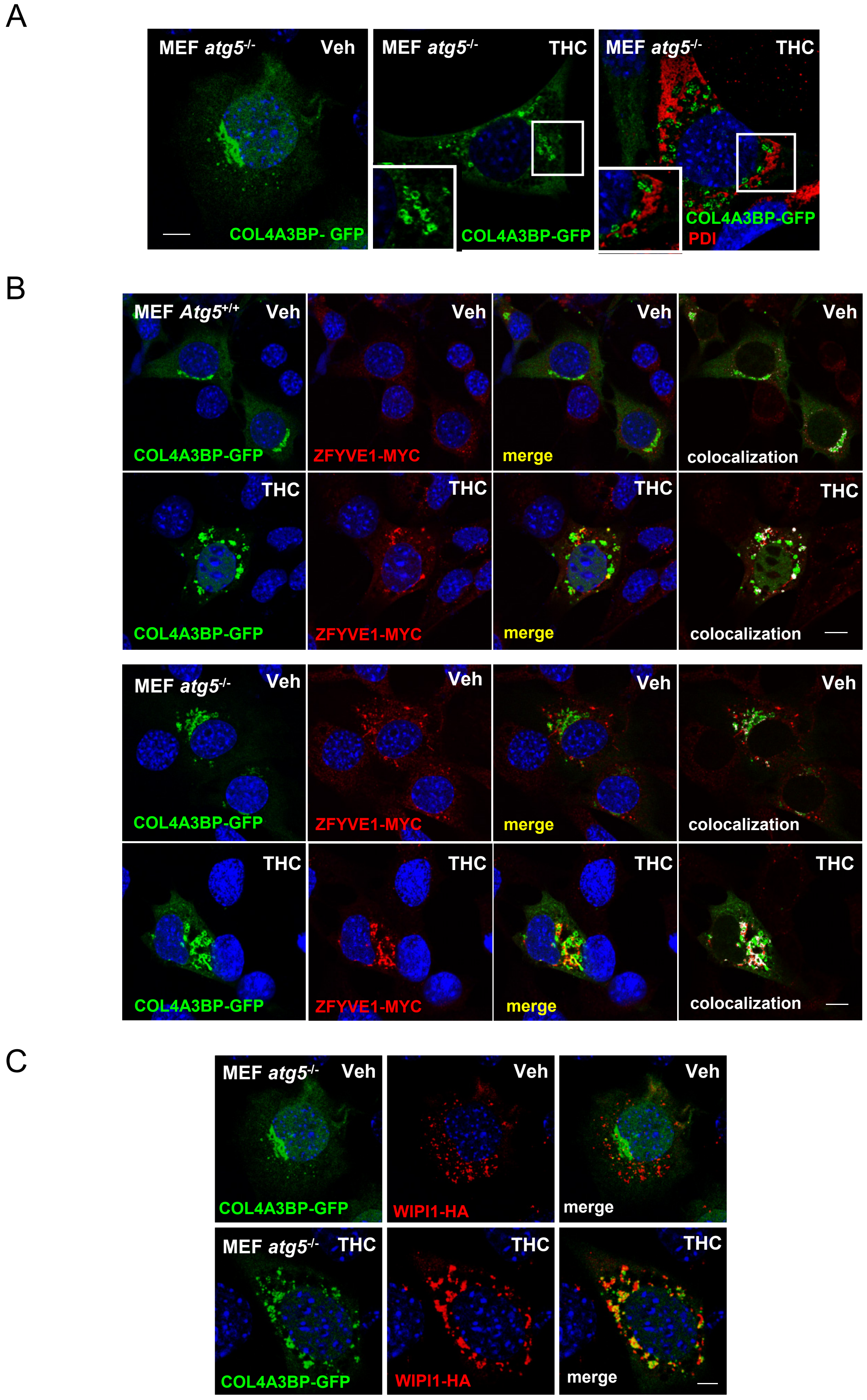

Figure S4. THC promotes the recruitment of COL4A3BP into omegasomes (**A**) Effect of THC (5 µM, 7 h) on the subcellular distribution of COL4A3BP-GFP in T-large antigen-immortalized *atg5*$^{-/-}$ MEFs. Note that COL4A3BP accumulates in ring-shaped vesicles located near the ER (n = 3; a representative experiment is shown). Bar = 20 µm. (**B**) Effect of THC (5 µM, 7 h) on the sub-cellular distribution of COL4A3BP-GFP and ZFYVE1/DFCP1-MYC in *Atg5*$^{+/+}$ and *atg5*$^{-/-}$ T-large antigen-immortalized MEFs (n=3; a representative experiment is shown). Bar = 20 µm. (**C**) Effect of THC (5 µM, 7 h) on the sub-cellular distribution of COL4A3BP-GFP and WIPI1-HA in *atg5*$^{-/-}$ T-large antigen-immortalized MEFs. Bar: 20 µm.

Hernández-Tiedra et al. Figure S5

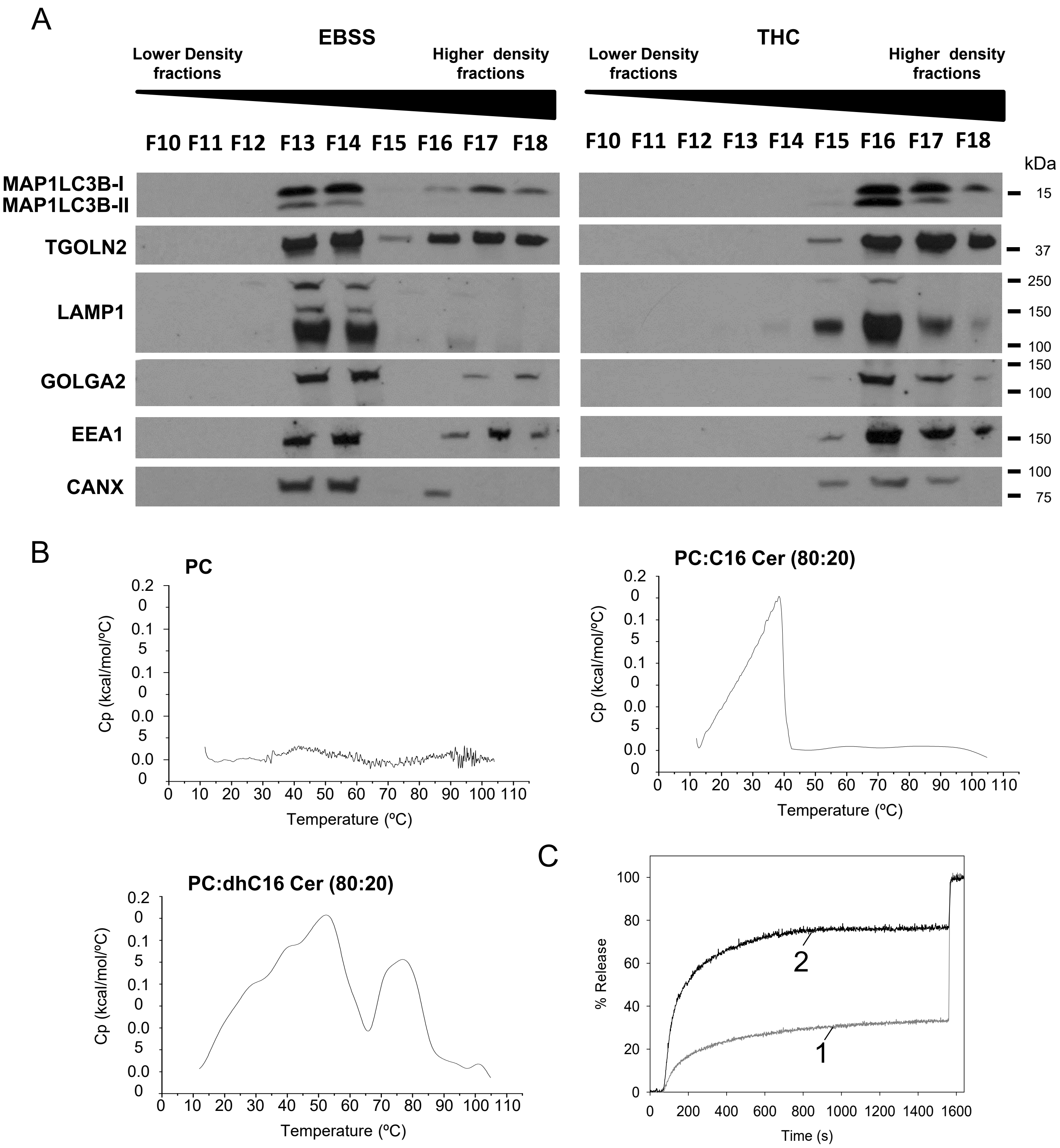

Figure S5. Dihydroceramides destabilize biological membranes. (**A**) Characterization of the presence of markers of different organelles on fractions obtained from U87MG cells incubated for 6 h with EBSS or THC (6 μM) and subjected to subcellular fractionation in an OptiPrep® gradient. EEA1 (early endosome antigen 1) (early endosome marker); CANX (calnexin) (ER marker), LAMP1 (lysosomal-associated membrane protein 1) (lysosomal marker); GOLGA2/GM130 (golgin A2) (cis-Golgi marker); TGOLN2/TGN38 (trans-golgi network protein 2) (trans Golgi marker). (**B**) Effect of C16 ceramides and C16 dihydroceramides on the gel-to-fluid phase transition of lipid membranes, measured by differential scanning calorimetry (DSC). (**C**) Release of vesicular aqueous contents induced by C16 ceramide and C16 dihydroceramide. Ceramides were generated by the action of sphingomyelinase on LUVs composed of 1, SM:PE:PC:Ch (1:1:1:1) (initial slope: 0.25 ± 0.030); 2, dhSM:PE:PC:Ch (1:1:1:1) (initial slope: 0.51 ± 0.041). (n = 3; *P* [Student's t-test] = 0.007). SM, sphingomyelin; dhSM, dihydrosphingomyelin; PE, phosphatidylethanolamine; PC, phosphatidylcholine; Ch, cholesterol.

Hernández-Tiedra et al. Figure S6

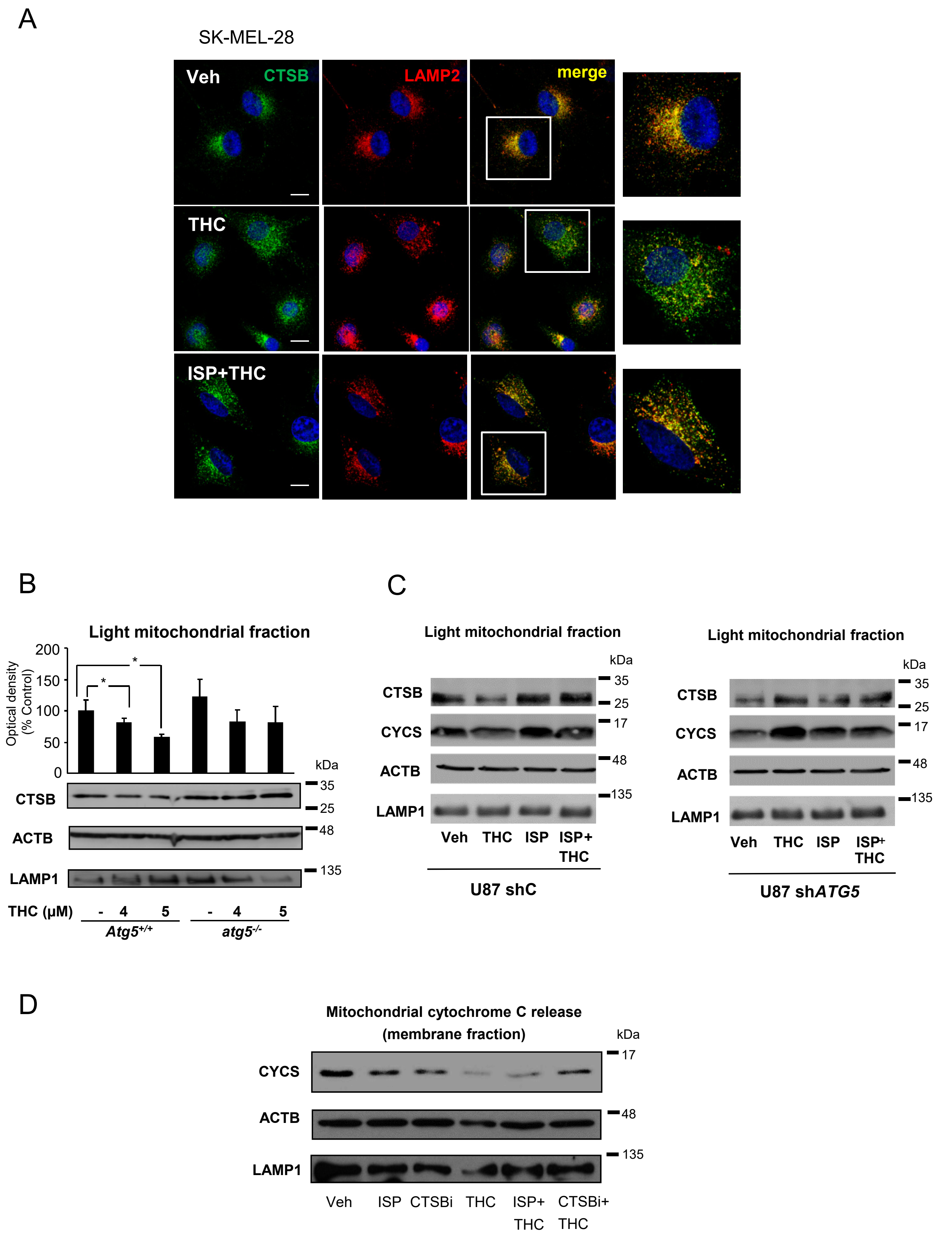

Figure S6. THC promotes lysosomal membrane permeabilization in a sphingolipid- and autophagy-dependent manner in cancer cells. (**A**) Effect of THC (4 μM, 18 h) and ISP-1 (5 μM) on CTSB and LAMP2 subcellular distribution (as determined by immunofluorescence) of SK-MEL-28 cells (n = 3). Bar: 20 μm. (**B**) Effect of THC (4 μM, 18 h) on CTSB distribution in the membrane-associated fraction of *Atg5*$^{+/+}$ or *atg5*$^{-/-}$ (autophagy-deficient) HRASV12/T-large-transformed MEFs (n = 4). Western blot analyses of a representative experiment are shown. LAMP1 is included as a control for the presence of membrane-associated proteins in the light mitochondrial fraction. Data correspond to the densitometric analysis of mature CTSB relative to the corresponding loading control and are expressed as the percentage of mature CTSB ± s.d. relative to *Atg5*$^{+/+}$ vehicle-treated cells (n = 4; *, $P < 0.05$ from vehicle-treated cells). (**C**) Effect of THC (4 μM, 16 h) and ISP-1 (5 μM) on CTSB and CYCS distribution in the membrane-associated fraction of control (shC) and sh*ATG5* U87MG cells. LAMP1 is included as a control for the presence of membrane-associated proteins in the light mitochondrial fraction (n=3; western blot analyses of a representative experiment are shown). (**D**) Effect of THC (4 μM), ISP-1 (5 μM) and CTSB inhibitor (CTSBi, 10 μM) on CYCS distribution in the membrane-associated fraction of U87MG cells. LAMP1 is included as a control for the presence of membrane-associated proteins in the light mitochondrial fraction (n = 3; western blot analyses of a representative experiment are shown).

Hernández-Tiedra et al. Figure S7

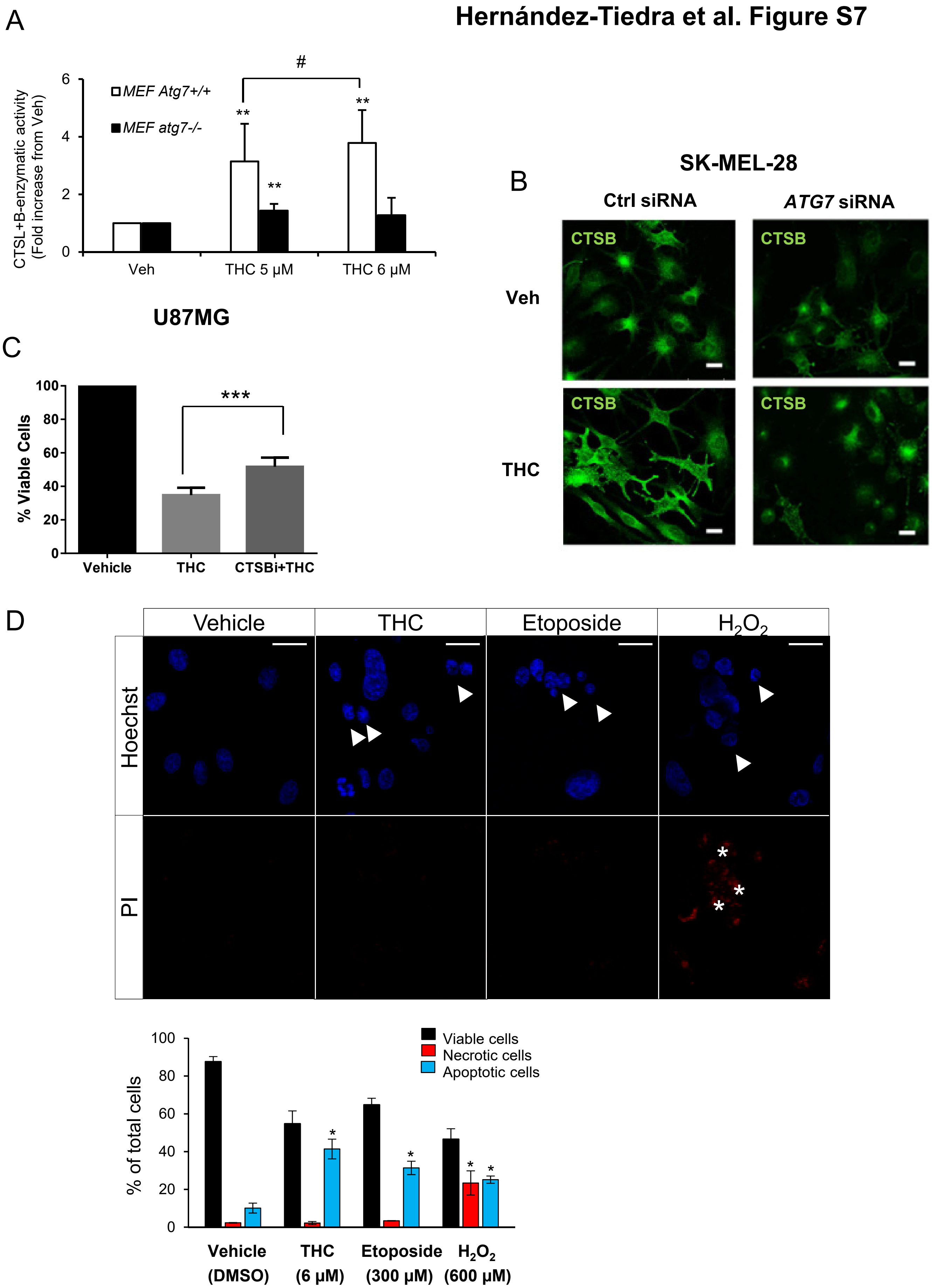

Figure S7. THC promotes lysosomal membrane permeabilization and induces apoptosis but not necrosis in cancer cells. (**A**) Effect of THC on the CTSL+ CTSB (cytosolic) enzymatic activity of T-large antigen-immortalized *Atg7*$^{+/+}$ and *atg7*$^{-/-}$ MEFs. (**B**) Effect of THC on the sub-cellular distribution of CTSB (green) of SK-MEL-28 melanoma cells transfected with siC or *ATG7*-selective siRNA (si*ATG7*). Note that genetic blockade of autophagy prevents the effects of THC on CTSB subcellular distribution. Bars: 20µm. (**C**) Effect of THC (6 µM, 24 h) and CTSBi (10 µM) on the number of viable cells (as estimated by the MTT test). Data are expressed as the percentage of viable cells relative to vehicle-treated cells (mean ± s.d, n = 7, ***, $P < 0.001$ from THC-treated cells). No significant differences were found between the number of CTSBi- and Veh-treated cells (as estimated by the MTT assay). (**D**) Effect of THC (6 µM, 16 h), etoposide (300 µM, 16 h) or H_2O_2 (600 µM, 16 h) on viability, apoptosis and necrosis (as determined by staining with propidium iodide and Hoechst 33342). Data are expressed as the percentage of viable, apoptotic and necrotic cells relative to the total number of nuclei in each field and correspond to 20 fields of 3 different wells for each experimental condition (mean ± s.e., n = 4, *, $P < 0.05$ from vehicle treated cells). Cells without pyknotic nuclei and PI staining were scored as viable. Cells with pyknotic nuclei (white arrows in microphotographs) were scored as apoptotic. Cells with PI^+ staining (asterisks in microphotographs) were scored as necrotic. THC and etoposide treatments only increased the percentage of apoptotic cells, whereas H_2O_2 treatment increased the percentages of both necrotic and apoptotic cells. Bars: 20 µm.

Hernández-Tiedra et al. Figure S8

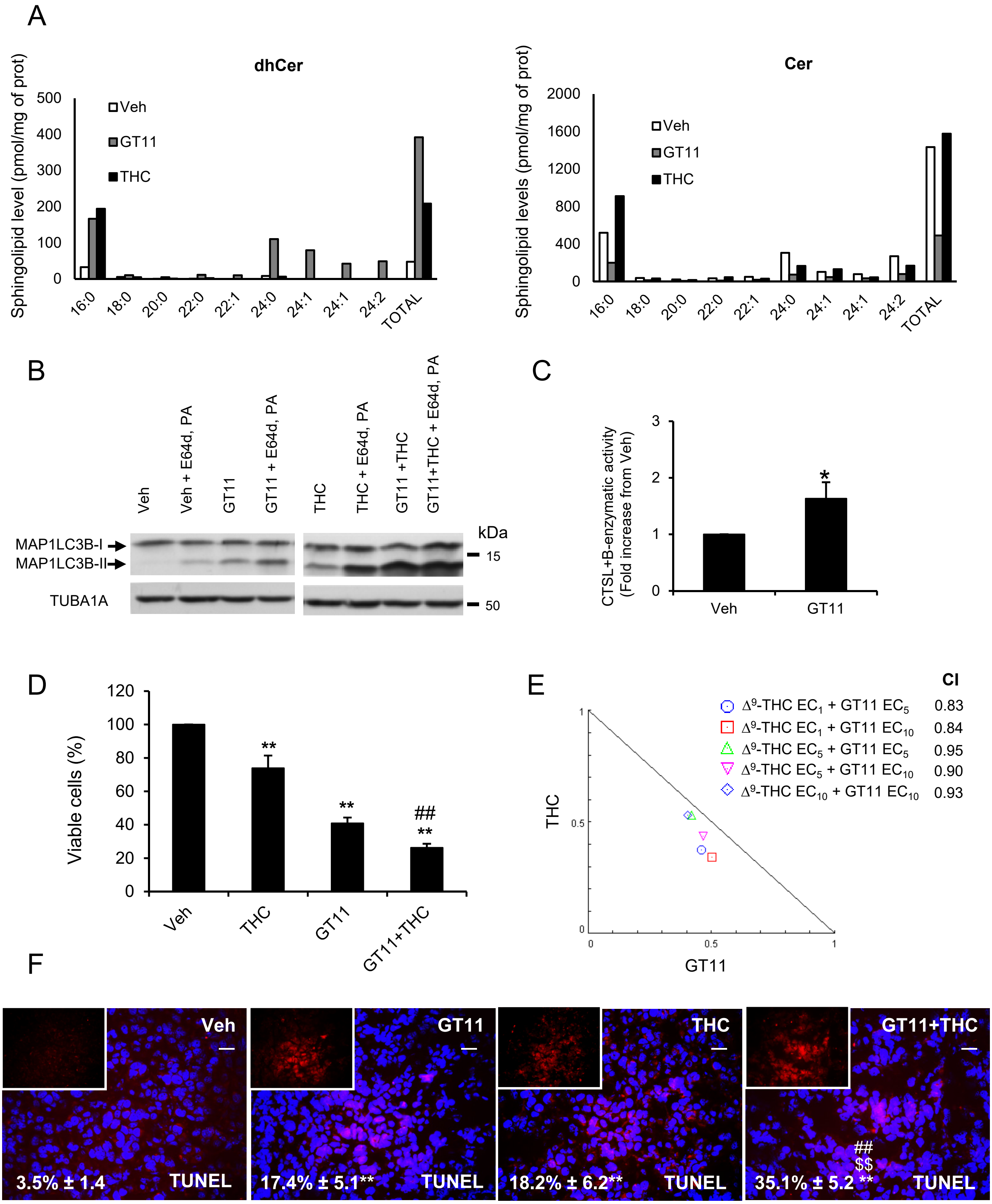

Figure S8. Treatment with GT11 induces accumulation of dihydroceramide, autophagy and cathepsin release in U87 cells, and the combination with THC increases its antitumoral effect on cancer cells. (**A**) Effect of THC (4 µM, 6 h), GT11 (3 µM, 6 h) and the combination of THC and GT11 on the levels of total ceramides and dihydroceramides found in total cell lysates of U87MG cells (n = 2; a representative experiment is shown). (**B**) Effect of THC (3.5 µM, 18 h), GT11 (3 µM, 18 h) and the combination of THC and GT11 on autophagy (as determined by MAP1LC3B lipidation) (n = 3, a representative experiment is shown). (**C**) Effect of GT11 (3 µM; 18 h) on the CTSL + CTSB (cytosolic) enzymatic activity of U87MG cells (n = 4; *, $P < 0.05$ from vehicle-treated cells). (**D**) Effect of THC (3 µM, 18 h), GT11 (3 µM, 18 h) and the combination of THC and GT11 on the viability of U87MG cells. (n = 4; **, $P < 0.01$ from vehicle-treated cells and $^{\#\#}$, $P < 0.01$ from GT11-treated cells). (**E**) Drug combination index analysis for THC and GT11. Doses used to perform the isobologram and confidence interval values are indicated in the figure and were obtained using the CompuSyn program. Analysis of the data showed that the combination of the 2 drugs produced a slight synergism. (**F**) Effect of THC (15 mg/kg), GT11 (7.5 mg/kg) or THC and GT11 on apoptosis (as determined by TUNEL). Values in the lower left corner correspond to the percentage of TUNEL-positive cells relative to the number of nuclei in each field, and correspond to 10 fields of 3 different tumors for each condition; expressed as the mean fold change ± s.d. Representative images from the different experimental conditions are shown. Bar: 20 µm.

Table S1. THC modifies the ceramide:dihydroceramide ratio in the microsomal fraction of U87MG cells.

**dhCer (pmol/mg prot)**						
	**Veh**	**THC**	**SD Veh**	**SD THC**	**p-value (THC vs Veh)**	
**C14**	0.40103365	1.74654393	0.39413193	1.65595175	0.09008192	
**C16**	6.17755474	17.1560462	4.48091293	12.017723	0.04047562	*
**C18**	0.13581785	0.29147932	0.17580421	0.5688066	0.51500947	
**C20**	0.16748377	0.39033193	0.21712274	0.72587634	0.46279104	
**C22**	0.6195158	1.4484358	0.45476856	1.02133874	0.07797108	
**C24**	2.79297033	8.03721893	1.97132723	4.02614415	0.00949994	***
**C24:1**	3.25312546	14.5117921	2.25703557	7.98792241	0.01552537	*
∑	13.5475016	43.5818482	8.35089505	24.7505499	0.02018489	*

**Cer (pmol/mg prot)**						
	**Veh**	**THC**	**SD Veh**	**SD THC**	**p-value (THC vs Veh)**	
**C14**	4.49372941	6.73671891	3.7465922	4.19228761	0.03898503	*
**C16**	44.5435044	47.55427	37.5253716	37.0219128	0.45611976	
**C18**	1.30490929	1.78798684	0.94779821	1.26825388	0.14878418	
**C20**	0.4988006	0.64521187	0.33549782	0.63385336	0.47030877	
**C22**	4.39498046	5.21387862	3.16498755	3.61736124	0.20242571	
**C24**	22.0746559	29.0883733	16.5990494	17.2627899	0.02792114	*
**C24:1**	32.0028416	40.2726149	19.9191298	22.7298907	0.03073715	*
∑	111.721154	134.318856	80.3544075	84.1220224	0.03763073	*

**Cer:dhCer**						
	**Veh**	**THC**	**SD Veh**	**SD THC**	**p-value (THC vs Veh)**	
**C16**	6.99176797	2.94813637	2.39597695	0.8029098	0.01465338	**
**C24**	9.13227619	3.81344839	4.40683817	1.16525066	0.041724	*
**C24:1**	10.5972204	2.96538217	2.25641975	0.74235059	0.00127305	***
∑	8.51732591	3.30197183	3.80112646	1.01003223	0.02506979	*

Effect of THC treatment (6 µM, 6 h) on the levels of the different molecular species of ceramides and dihydroceramides (as determined by liquid chromatography-tandem mass spectrometry) and in the ceramide to dihydroceramide ratio found in the microsomal fraction of U87MG cells. Column 1 shows the molecular specie of sphingolipid analyzed. Columns 2 (Veh) and 3 (THC) show the mean; columns 4 (SD Veh) and 5 (SD THC) the standard desviation (s.d.) and column 6 (p value [THC vs Veh]) the p value (student t test) of 5 independent experiments. Column 7: *, $P < 0.05$; **, $P < 0.01$; and ***, $P < 0.001$ from vehicle-treated cells. dhCer, dihydroceramide; Cer, ceramide.

SUPPLEMENTARY MATERIALS AND METHODS

Genetic knockdown by small interfering RNA.

SK-MEL28 cells were transfected with siRNA duplexes using Dharma-FECT 1 Transfection reagent (Dharmacon, T-2001-03). Twenty-four h after transfection, cells were trypsinized and seeded at a density of 5000 cells/cm^2. Human siRNAs to knock down ATG7 were purchased from Dharmacon as a SMARTpool (Dharmacon, L-020112-00-0005). These reagents combine 4 SMART selection designed siRNAs into a single pool, which guarantees an efficiency of silencing of at least 75%. The double stranded siRNA in all cases were designed and synthesized by Dharmacon. The nontargeted control (5'-UUCUCCGAACGUGUCACGU- 3'), was synthesized by Eurogentec (Liege, Belgium).

Differential scanning calorimetry (DSC).

Both lipid suspension and buffer were degassed before being loaded into the sample or reference cell of a VP-DSC Microcalorimeter (MicroCal; Northampton, MA, USA). Three heating scans, at 45ºC/h were recorded for each sample. Data treatment was performed using the software ORIGIN (MicroCal) provided with the calorimeter.

Immunoprecipitation.

For immunoprecipitation experiments, cells were lysed in a buffer containing 40 mM HEPES, pH 7.5, 120 mM NaCl, 1 mM EDTA, 10 mM sodium pyrophosphate, 10 mM sodium glycerophosphate, 50 mM sodium fluoride, 0.5 mM sodium orthovanadate, 0.3% CHAPS (Sigma-Aldrich, C3023). Briefly, lysate (1–4 mg) was pre-cleared by incubating with 5–20 µl of protein G-Sepharose (Sigma-Aldrich, GE17-0618-01). The

lysate extracts were then incubated with 5–20 µl of protein G-Sepharose conjugated to 5–20 µg of the antibody (the antibody was previously covalently coupled to protein G-Sepharose using dimethyl pimelimidate, [Sigma-Aldrich, D8388]). Immunoprecipitations were carried out overnight on a rotating wheel. The immunoprecipitates were washed 4 times with lysis buffer, followed by 2 washes with HEPES buffer (25 mM HEPES, pH 7.5, 50 mM KCl). Then the immunoprecipitates were resuspended in 30 µl of sample buffer (not containing 2-mercaptoethanol), filtered through a 0.22-µm pore size Spin-X filter (Sarstedt, 83 1826 001) and 2-mercaptoethanol (Sigma-Aldrich, M3148) was finally added to a concentration of 1% (v/v). Samples were subjected to electrophoresis and immunoblot analysis. Western blot analysis was performed following standard procedures. Densitometric analysis was performed with Quantity One software (Bio-Rad; Hercules, CA, USA).

Hoechst staining for apoptosis detection.

U87 glioma cells cultured on 12-well plates were treated with THC (6 µM), etoposide (300 µM; Sigma-Aldrich, E1383) or H_2O_2 (600 µM; Sigma-Aldrich, 516813) during 16 h and subsequently stained (with the cells alive) with Hoechst 33342 and propidium iodide (PI; Roche Life Science, 11348639001) at a final concentration of 1 µg/ml and 0.2 µg/ml, respectively. After incubation for 20 min, nuclear morphology and PI staining was observed using an Eclipse TS100 inverted microscope (Nikon, Amstelveen, The Netherlands) and a 40 x dry objective. For each treatment, cells were analyzed in at least 4 subfields of each culture. Cells without chromatin condensation and PI staining were scored as viable cells, whereas those with pyknotic nuclei were scored as apoptotic cells. Cells with PI-positive (+) staining were scored as necrotic cells.
